# Oscillatory Behaviour of Ni Supported on ZrO_2_ in the Catalytic Partial Oxidation of Methane as Determined by Activation Procedure

**DOI:** 10.3390/ma14102495

**Published:** 2021-05-12

**Authors:** Daniela Pietrogiacomi, Maria Cristina Campa, Ida Pettiti, Simonetta Tuti, Giulia Luccisano, Leandro Ardemani, Igor Luisetto, Delia Gazzoli

**Affiliations:** 1 Chemistry Department, Sapienza University of Rome, P.le Aldo Moro 5, 00185 Rome, Italy; ida.pettiti@uniroma1.it (I.P.); giulia.luccisano@uniroma1.it (G.L.); leandrodoc@yahoo.com (L.A.); delia.gazzoli@uniroma1.it (D.G.); 2 Institute for the Study of Nanostructured Materials (ISMN), National Research Council (CNR), Sapienza University of Rome, P.le Aldo Moro 5, 00185 Rome, Italy; mariacristina.campa@cnr.it; 3 Science Department, Roma Tre University, Via della Vasca Navale 79, 00146 Rome, Italy; simonetta.tuti@uniroma3.it; 4 Italian National Agency for New Technologies, Energy and Sustainable Economic Development (ENEA), Casaccia Research Centre, Via Anguillarese 301, 00123 Rome, Italy; igor.luisetto@enea.it

**Keywords:** supported Ni/ZrO_2_ catalysts, Ni metal particles, methane partial oxidation, activation procedure, heterogeneous catalysis

## Abstract

Ni/ZrO_2_ catalysts, active and selective for the catalytic partial oxidation of methane to syngas (CH_4_-CPO), were prepared by the dry impregnation of zirconium oxyhydroxide (Z_hy_) or monoclinic ZrO_2_ (Z_m_), calcination at 1173 K and activation by different procedures: oxidation-reduction (*ox-red*) or direct reduction (*red*). The characterization included XRD, FESEM, in situ FTIR and Raman spectroscopies, TPR, and specific surface area measurements. Catalytic activity experiments were carried out in a flow apparatus with a mixture of CH_4_:O_2_ = 2:1 in a short contact time. Compared to Z_m_, Z_hy_ favoured the formation of smaller NiO particles, implying a higher number of Ni sites strongly interacting with the support. In all the activated Ni/ZrO_2_ catalysts, the Ni–ZrO_2_ interaction was strong enough to limit Ni aggregation during the catalytic runs. The catalytic activity depended on the activation procedures; the *ox-red* treatment yielded very active and stable catalysts, whereas the *red* treatment yielded catalysts with oscillating activity, ascribed to the formation of Ni^δ+^ carbide-like species. The results suggested that Ni dispersion was not the main factor affecting the activity, and that active sites for CH_4_-CPO could be Ni species at the boundary of the metal particles in a specific configuration and nuclearity.

## 1. Introduction

The global energy demand is expected to rise by 30% between today and 2040. In this scenario, the use of natural gas as an energy source is expected to increase by 45% in the next 20 years, according to the World Energy Outlook 2017—International Energy Agency [1]. The conversion of natural gas, mainly constituted by methane, into highly valuable products has become challenging [1,2,3,4]. The catalytic conversion of methane to syngas is an attractive research area as syngas is a building block for valuable chemicals and liquid fuels [5,6,7]. The currently adopted large-scale process to produce syngas is CH_4_ steam reforming [8,9], which is energetically expensive due to its endothermic characteristics. Conversely, the catalytic partial oxidation of methane (CH_4_-CPO) is a valid alternative because it is mildly exothermic and can produce syngas in a ratio (H_2_/CO = 2) suitable for methanol or Fischer–Tropsch synthesis [5,6,7,10,11,12,13].

Catalytic systems based on noble (Rh, Pt and Ir) or nonnoble metals (Ni, Co and Fe) supported on various oxides have been studied for the CH_4_-CPO reaction [4,5,11,14]. By consensus, nickel supported on oxides is considered a promising catalyst owing to the high methane conversion, high CO and H_2_ selectivities and low cost [11,14,15,16,17,18,19,20], but it suffers from deactivation. Many studies have shown that deactivation arises from the sintering of Ni particles and/or coke deposition, both depending on metal particle and support features [11,14,21,22,23,24]. The choice of support is fundamental, as the metal–support interaction can favour the formation of peculiar active sites at the metal–support boundaries [25,26].

Zirconium oxide is widely used as a support because of its high thermal stability and characteristic textural properties that can be tailored according to different preparation methods and thermal treatments, yielding the formation of monoclinic, metastable tetragonal or mixed tetragonal-monoclinic ZrO_2_ phases [27,28,29]. Many papers have extensively investigated Ni-supported ZrO_2_ systems for syngas production focussing on the support preparation methods and on the deposition of Ni precursors to obtain metal particle sizes suitable for high catalytic performances and low carbon deposition [11,14,30,31,32,33,34,35,36]. By contrast, the effect of ZrO_2_ modifications on the metal dispersion and on the structure–activity relationship has not been clearly established thus far. Studies on the interaction between Ni clusters and ZrO_2_ polymorphs have reported that monoclinic ZrO_2_ performs better than tetragonal and cubic forms both for cluster dispersion and aggregation inhibition [37,38]. Furthermore, the effect of the activation procedures of catalyst precursors on the Ni metal particle features has not been deeply analysed, although it is generally recognised to affect the strength of the metal–support interaction [39].

In this paper, we studied the catalytic performances for the CH_4_-CPO of nickel-supported systems in which the support was ZrO_2_ in the monoclinic modification. The thermal stability of the monoclinic phase up to about 1400 K, able to prevent changes induced by the occurrence of hot spots during CH_4_-CPO reaction, and the chemical inertness between NiO and ZrO_2_ are expected to assure catalyst stability. Ni/ZrO_2_ catalysts were prepared by the impregnation of two different starting materials (a high-surface-area zirconium oxyhydroxide, Z_hy_, and a low-surface-area monoclinic zirconium oxide, Z_m_) and were activated following two different procedures (oxidation–reduction treatment, *ox-red,* or direct reduction treatment, *red*). The objective of this study was to clarify the effect of the starting material and of the reductive activation procedure on the nature of the active phase and on the catalytic behaviour. To this aim, we compared the catalytic behaviours and features of Ni/ZrO_2_ catalysts with those of bare monoclinic ZrO_2_ and of an unsupported Ni powder sample. Materials were characterized by various techniques, including atomic absorption spectroscopy (AAS), X-ray diffraction (XRD), specific surface area (BET method) and pore volume analysis, field-emission scanning electron microscopy (FESEM), temperature-programmed reduction (TPR), and in situ transmission Fourier-transform infrared (FTIR) and Raman spectroscopies. The correlation between the characterization and catalytic activity results allowed some hypotheses to be formulated on the active sites for the CH_4_-CPO and on the consequent oscillating catalytic behaviour.

## 2. Materials and Methods

### 2.1. Materials

Zirconium oxyhydroxide (Z_hy_) was prepared via precipitation from a ZrOCl_2_ solution with ammonia. After separation, the solid was washed with water until a Cl^−^-negative test (no opalescence in the liquid after addition of AgNO_3_). The test was conducted on the washing water and on small portions of solid removed from the batch and dissolved in dilute HNO_3_. After washing, the solid was dried at 383 K for 24 h. A portion of this material was calcined at 1173 K (heating rate: 0.25 K·min^−1^) for 5 h, indicated as Z_m_ (m stands for monoclinic).

NiO/ZrO_2_ catalyst precursors (about 2 or 5 Ni wt.%) were prepared by the dry impregnation of Z_hy_ or Z_m_ material with an Ni(NO_3_)_2_ aqueous solution, and they were dried at 383 K and subsequently calcined at 1173 K for 5 h; the heating rate was 0.25 K·min^−1^ for the Z_hy_-based samples and was 5.0 K·min^−1^ for those based on Z_m_. The adoption of such a slow heating ramp (0.25 K·min^−1^) for the Z_hy_-based samples was necessary to obtain crystalline ZrO_2_ with the highest possible surface area, avoiding a marked local increase in temperature due to the exothermicity of the crystallization process. NiO/ZrO_2_ catalyst precursors were stored in plastic vials at room temperature in air and were labelled as *x*NiO/Z*_a_*, where *x* indicates the Ni content (Ni wt.%) and Z*_a_* specifies the starting material used for the impregnation, Z_hy_ or Z_m_.

**Scheme 1:**
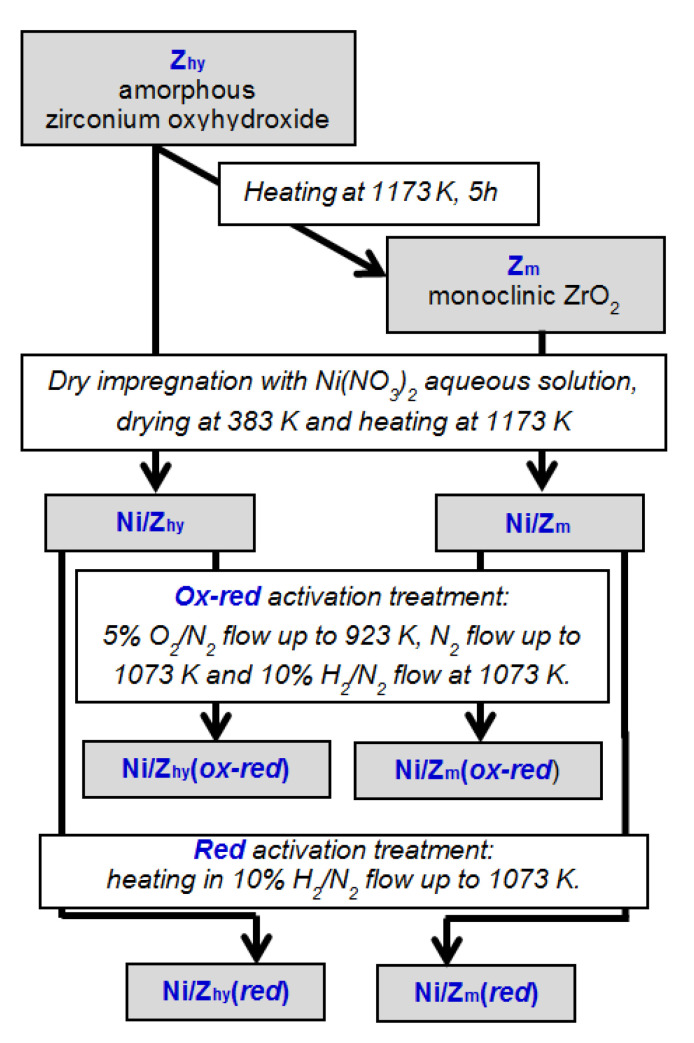
Preparation procedures of Ni/ZrO_2_ catalysts.

Ni/ZrO_2_ catalysts were obtained by the in situ reduction of precursors following different activation procedures: (i) oxidation in a 5% O_2_/N_2_ flow up to 923 K for 1 h, purging with N_2_ up to 1073 K, and reduction in a 10% H_2_/N_2_ flow at 1073 K for 1 h (*ox-red* activation); (ii) direct reduction in a 10% H_2_/N_2_ flow up to 1073 K for 1 h (*red* activation). Samples were named xNi/Z_a_ (*ox-red*) and xNi/Z_a_ (*red*), respectively. The catalyst preparation procedures are sketched in Scheme 1.

Polycrystalline nickel oxide was obtained by the slow decomposition of Ni(CH_3_COO)_2_ in air at 1173 K (heating rate: 0.25 K·min^−1^) for 5 h.

### 2.2. Characterization

The Ni content of catalysts was determined by AAS (SpectrAA 220, Varian Australia Pty Ltd., Mulgrave, Australia). X-ray diffraction (XRD) patterns were obtained with a Philips PW 1729 diffractometer (Malvern Panalytical Ltd., Malvern, UK) using Cu Kα (Ni-filtered) radiation in a 2θ range of 10–70° (step size: 0.02°; time per step: 1.25 s). The mean crystallite diameter (*d*) was calculated by the Scherrer equation [40] as *d* = *K*λ/βcosθ, where *K* is a shape constant equal to 0.9, λ is the X-ray wavelength used, and β is the effective linewidth (FWHM) of the observed X-ray reflection, obtained by a curve-fitting procedure after Warren’s correction for instrumental broadening and background subtraction.

Specific surface area (BET method) and textural properties were determined by the adsorption/desorption of N_2_ at 77 K using a Micromeritics ASAP 2010 Analyzer (Norcross, GA, USA) after sample outgassing at 473 K for 2 h via thermally controlled heating mantles, up to a residual pressure lower than 0.8 Pa. The pore distribution was determined by the BJH method [41] from the adsorption isotherm. The total pore volume was obtained by the rule of Gurvitsch [42]. The uncertainty was ±0.5 m^2^·g^−1^ for the specific surface area values and ±0.005 cm^3^·g^−1^ for the total pore volume values.

Field-emission scanning electron microscopy (FESEM) images were obtained by using an AURIGA Zeiss 405 HR-FESEM instrument (Oberkochen, Baden-Württemberg**,** Germany), equipped with an energy-dispersive X-ray spectroscopy (EDXS) Bruker apparatus for elemental detection, which is able to discriminate particles with a minimum diameter of about 5 nm.

To evaluate the Ni dispersion, D (percent ratio of the exposed Ni atoms to the total Ni atoms), FESEM images were processed using the free ImageJ software version 1.53h (National Institutes of Health, Bethesda, MD, USA) [43] to measure the particle diameters, d_i_. The particle diameter distribution was established by measuring all the particles observed in at least 5 images of each sample. The error in the particle size measurement due to the pixel dimensions of FESEM images (depending on the resolution at different magnifications) was ≤10%. The metal dispersion was derived according to [44] using the approximation of spherical particles, taking into account the relationship between D and the mean particle diameter, D = 100·6 (*v*_Ni_/*a*_Ni_)/d_VA_, where d_VA_ = Σ_i_n_i_d_i_^3^/Σn_i_d_i_^2^ is the volume-area mean diameter, *v*_Ni_ is the volume of a Ni atom in bulk metal (0.01095 nm^3^), and *a*_Ni_ is the area occupied by a surface Ni atom (0.0651 nm^2^).

Temperature-programmed reduction (TPR) experiments were performed using a Thermo Scientific TPDRO1100 instrument (Waltham, MA, USA). The H_2_-TPR analysis was conducted by flowing a 5% H_2_/Ar mixture (10 cm^3^·min^−1^) through the sample (100 mg) from 323 to 1073 K (heating rate 10 K·min^−1^) and keeping the sample at 1073 K for 1 h. The H_2_ consumption was measured by a TCD detector, calibrated by the reduction of a known amount of CuO (99.99% purity; Sigma Aldrich, Saint Louis, MO, USA). Before flowing into the TCD, the H_2_O produced during the reduction was removed by a soda lime trap.

Raman spectra were recorded at room temperature, in back-scattering geometry, with an inVia Renishaw micro-Raman spectrometer (Wotton-under-Edge, Gloucestershire, UK), using the 488.0 nm emission line from an Ar ion laser as the exciting source. The power of the incident beam was about 5 mW. Repeated accumulations (10 or 20 scans × 10 s) were generally acquired on at least four regions of each sample using 20× or 5× objectives to check the sample homogeneity. Spectra were calibrated using the 520.5 cm^−1^ line of a silicon wafer. Spectra processing included baseline removal and curve fitting using a Gauss–Lorentz cross-product function by Peakfit 4.12 (Systat Software Inc., San Jose, CA, USA, 2007).

Transmission FTIR spectra were recorded with a Perkin Elmer Frontier spectrometer (Milano, Italy), equipped with a MCT detector operating with a resolution of 4 cm^−1^. The powdered sample, crushed and pelleted (pressure: 1.5 × 10^4^ kg·cm^−2^) in a self-supporting wafer of about 15 mg·cm^−2^, was inserted in a stainless-steel reactor equipped with CaF_2_ windows. The reactor, connected to a flow apparatus, allowed spectra to be recorded during thermal treatments up to 773 K in an oxidative (5% O_2_/N_2_) or reductive (10% H_2_/N_2_) flow to simulate the catalytic activation treatment. At a given temperature, surface species resulting from the various treatments were determined by subtracting reference spectra from those recorded after specific treatments. The reference spectra were collected in flowing helium, on samples heated up to 773 K and cooled down to the desired temperature (298–773 K).

### 2.3. Catalytic Activity

The CH_4_-CPO reaction was studied in a flow apparatus at atmospheric pressure. The feeding section included independent mass flow controller meters (MKS mod. 1259, driven by a four-channel unit MKS mod. 647C) and a glass ampoule for gas mixing before entering the reactor. The fixed-bed tubular reactor was made of two coaxial quartz tubes (i.d. 20 and 10 mm) to allow preheating of the feed gas. The temperature of the catalytic bed was monitored by a K-type thermocouple, located in a quartz tube (i.d. 4 mm) concentric to the reactor. High-purity gas mixtures (CH_4_/N_2_, O_2_/N_2_ and H_2_/N_2_ from Rivoira gas, Milano, Italy), and pure N_2_ (SOL) were used without further purification. Reactants and products were analysed by a Varian Micro-GC CP-4900 gas chromatograph (Agilent, Santa Clara, CA, USA) equipped with two columns (10 m Molsieve 5A BF, for H_2_, O_2_ and CO; 10 m Poraplot Q, for CH_4_, CO_2_ and H_2_O) and TCD detectors. Sample masses of 50 mg for ZrO_2_ and NiO/ZrO_2_ and 3.0 mg for unsupported NiO (nickel amount comparable to that contained in 50 mg of a catalyst with 5 wt.% Ni) were deposited on a ceramic wool bed in the reactor. The height of the catalytic bed in the reactor was ≤1 mm. Before catalytic measurements of Z_m_, unsupported NiO and NiO/ZrO_2_ catalysts were submitted to the in situ reduction procedure, *ox-red* or *red*, as specified in the Materials section (Section 2.1.).

Typical catalytic runs consisted of steady-state measurements with a mixture of 2% CH_4_, 1% O_2_, and N_2_ as balance, in the temperature range of 1023–723 K, leaving the catalysts in a continuous flow of the reactant mixture. The temperature was changed in a random sequence, maintaining a constant temperature for about 15 min (three consecutive Micro-GC analyses). To check the activity reproducibility, some temperature values were explored two or more times in the same run. For each catalyst, up to four runs were carried out by applying an *ox-red* activation treatment between two consecutive runs. The stability of the activity was tested in ad hoc runs following the reaction at a set temperature as a function of time on stream up to 5 h. The total flow rate was 150 cm^3^ (STP) min^−1^ (space velocity: 1.8·10^5^ NL·kg_cat_^−1^·h^−1^; contact time τ = 9 ms). At constant temperature, the linear increase in conversions at increasing contact time (τ) indicated that, under our experimental conditions, the reaction was under kinetic control without external diffusion effects (Figure S1 in the Supplementary Material). All experiments yielded a satisfactory carbon, hydrogen and oxygen balance (100 ± 5%). Samples after catalytic activity tests were named *used* catalysts.

The catalytic activity and selectivity for the CH_4_-CPO reaction (CH_4_ + 1/2O_2_ → CO + 2H_2_) were discussed considering the simultaneous occurrence of methane total combustion (CH_4_ + 2O_2_ → CO_2_ + 2H_2_O), and the water gas shift (WGS) reaction (CO + H_2_O → CO_2_ + H_2_). The percent CH_4_ conversion was calculated as 100 (CH_4_ molecules consumed)/(CH_4_ molecules injected). The percent H_2_ yield was calculated as 100 (H_2_ molecules produced)/(1/2 CH_4_ molecules injected), and CO or CO_2_ yields were calculated as 100 (CO or CO_2_ molecules produced)/(CH_4_ molecules injected). The H_2_ selectivity was calculated as 100 (H_2_ molecules produced)/(1/2 CH_4_ molecules converted). The CO selectivity was calculated as 100 (CO molecules produced)/(CH_4_ molecules converted). The rate of H_2_ production (R_H2_/molecules s^−1^·g^−1^) was calculated from H_2_ molecules produced in experiments in which the conversion never exceeded 30%. The rate was correlated to the number of exposed Ni atoms per gram of catalyst (N_Ni(exp)_), obtained according to the expression N_Ni(exp)_ = (D·w_Ni_·N_A_)/(10,000·M_Ni_), where D is the Ni dispersion value, w_Ni_ is the Ni loading (wt.%), N_A_ is Avogadro’s number and M_Ni_ is the Ni molar mass (58.69 g·mol^−1^).

## 3. Results and Discussion

### 3.1. Characterization

#### 3.1.1. Zirconium Oxide Support

**Figure 1:**
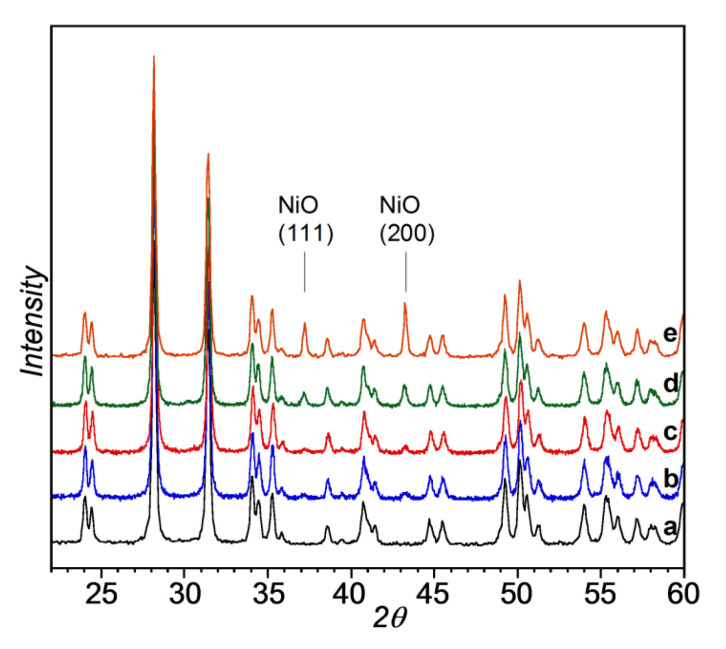
XRD patterns of monoclinic ZrO_2_ and NiO/ZrO_2_ catalyst precursors. (**a**) Z_m_; (**b**) 1.8NiO/Z_m_; (**c**) 1.7NiO/Z_hy_; (**d**) 4.8NiO/Z_hy_; (**e**) 5.1NiO/Z_m_.

Amorphous zirconium oxyhydroxide, Z_hy_, heated at 1173 K transformed into monoclinic ZrO_2_, Z_m_, as shown by XRD (JCPDF card 37-1484) (Figure 1a). The Z_m_ average crystallite size determined by the Scherrer equation [41] was 49 nm; the (−111) reflection at 28.4° was considered for the fitting procedure. Specific surface area measurements and textural analysis showed that Z_hy_ was a micro-mesoporous material, with a specific surface area of 360 m^2^·g^−1^, and that monoclinic ZrO_2_, Z_m_, was a meso-macroporous material, with a specific surface area of 5.6 m^2^·g^−1^ (Table 1).

**Figure 2:**
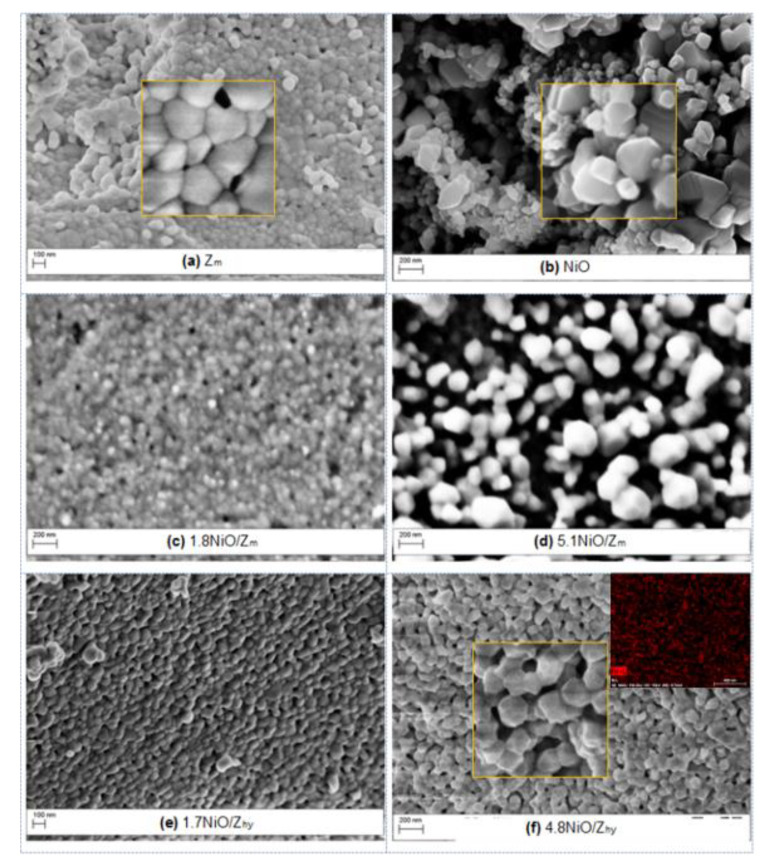
FESEM images of monoclinic ZrO_2_, unsupported NiO and NiO/ZrO_2_ catalyst precursors. Magnifications of selected areas are shown in the insets of (**a**), (**b**) and (**f**). The inset with red dots in (**f**) refers to the Ni EDX elemental map.

FESEM micrographs of the Z_m_ support revealed ZrO_2_ particles having sizes in the range of 30–100 nm (Figure 2a and Table 2).

#### 3.1.2. NiO/ZrO_2_ Catalyst Precursors

All the NiO/ZrO_2_ samples (NiO/Z_hy_ and NiO/Z_m_) heated at 1173 K, in addition to the reflection of monoclinic ZrO_2_, showed peaks at 2θ values of 37.3° and 43.3°, corresponding to the (111) and (200) crystallographic planes of cubic NiO (JCPDF card 4-0835), (Figure 1, patterns b–e). For all the NiO/ZrO_2_ samples, the NiO average crystallite sizes, determined by the (200) reflection (Scherrer equation [41]), ranged from 22 to 36 nm and was smaller than those of unsupported NiO (70 nm, Table 1), suggesting that an interaction between NiO and the support positively affected the crystallite sizes.

FESEM micrographs of unsupported NiO mainly revealed octahedral truncated particles (inset in Figure 2b) with sizes in the range of 20–400 nm (Figure 2b and Table 2). The images of the NiO/Z_m_ samples revealed NiO particles (EDX analysis) with sizes within the range exhibited by unsupported NiO, about 40–60 nm for the 1.8NiO/Z_m_ sample and 80–300 nm for the 5.1NiO/Z_m_ sample (Figure 2c,d; Table 2). In particular, in the latter sample, NiO-supported particles showed truncated octahedral shapes similar to those of unsupported NiO particles. The NiO particles imaged by FESEM were larger than those determined by XRD, indicating that, on the zirconia surface, NiO was in the form of crystallite aggregates.

The nitrogen adsorption/desorption Type IV isotherms (Figure S2a) showed H1 hysteresis loops characteristic of adsorbents with a narrow distribution of uniform pores. The surface area and total pore volume values of NiO/Z_m_ samples were lower than those of the Z_m_ support (Table 1), due to the decrease in the meso- and macroporosity, suggesting that some of the NiO particles partially occluded the pores. Accordingly, pore size distribution plots exhibited a mesoporous texture more abundant for Z_m_ than for the NiO/ZrO_2_ catalyst precursors (Figure S2b).

In the NiO/Z_hy_ samples, although EDX analysis detected a homogeneous distribution of Ni species, and NiO crystallite sizes estimated by XRD were above the size detection limit of FESEM (5 nm), no NiO particles were imaged (Figure 2e,f). This fact could be due to (i) the low contrast between supported NiO particles and the zirconia support, and/or (ii) the location of the NiO particles in the ZrO_2_ interparticle voids, formed during the calcination of Z_hy_ impregnated with Ni(NO_3_)_2_. The similarity of surface area and total pore volume values between NiO/Z_hy_ samples and the Z_m_ support (Table 1) suggests that, for NiO/Z_hy_ samples, pore occlusion of ZrO_2_ by NiO did not occur, supporting the hypothesis of small NiO particles. A closer inspection of the 4.8NiO/Z_hy_ FESEM image (inset in Figure 2f) indicated particles with truncated octahedral morphologies, unlike the roundish particles of the Z_m_ support but similar to those of unsupported NiO particles. This result suggests that, during the calcination treatment, the NiO phase spread as a raft-like layer on the Z_hy_ support. This intimate contact between the two oxide phases could account for the low contrast in the FESEM images [45].

**Figure 3:**
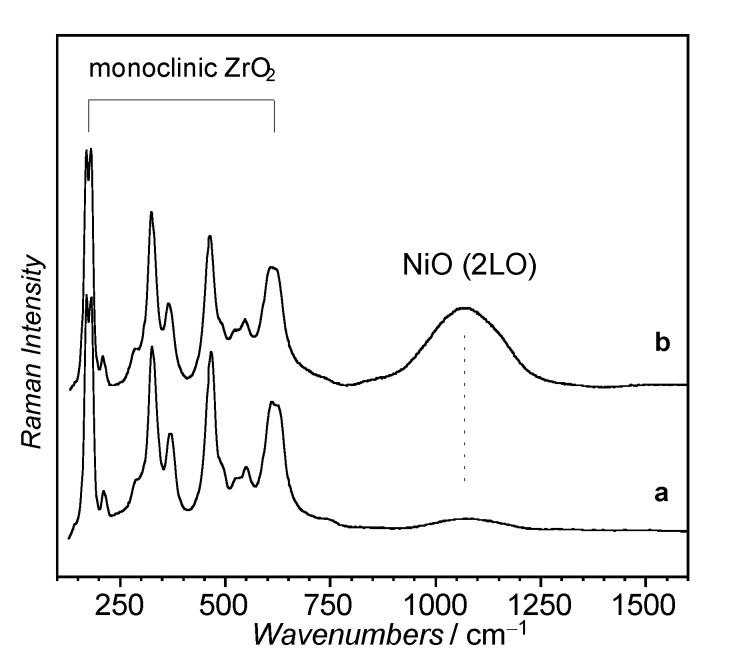
Raman spectra of catalyst precursors. (**a**) 4.8NiO/Z_hy_; (**b**) 5.1NiO/Z_m_.

Raman spectra confirmed the presence of NiO both in the NiO/Z_m_ and NiO/Z_hy_ samples (Figure 3). NiO showed Raman bands at about 420 and 560 cm^−1^ (one-phonon, 1P: TO and LO modes, respectively) and at about 730, 906 and 1090 cm^−1^ (two-phonon, 2P: 2TO, TO + LO and 2LO modes, respectively) [46]. With the decrease in particle size, the 1P bands became more pronounced due to defects, whereas the 2P bands broadened and decreased in intensity [47]. For both 5.1NiO/Z_m_ and 4.8NiO/Z_hy_ samples, while the 1P and 2P modes in the range of 200–750 cm^−1^ were completely obscured by those of monoclinic ZrO_2_, the broad band at about 1090 cm^−1^ (2LO mode) clearly indicated the presence of the NiO phase (Figure 3). The much higher intensity of the 2LO band in the 5.1NiO/Z_m_ sample than in the 4.8Ni/Z_hy_ sample was consistent with the presence of larger NiO particles in the former than in the latter.

Temperature-programmed reduction (TPR) was applied to study the reducibility of NiO species, which is expected to depend on the strength of interaction with the support. In all the samples, NiO species were completely reduced to Ni metal in the temperature range of 473–923 K (total H_2_ consumption corresponding to about 2 e/Ni, Table 3).

**Figure 4:**
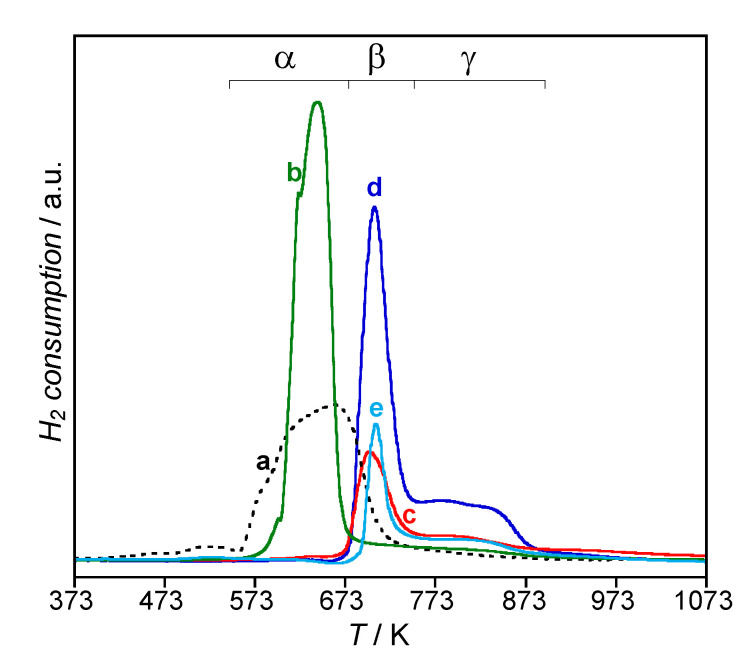
TPR profiles for NiO and NiO/ZrO_2_ catalyst precursors. (**a**) Unsupported NiO; (**b**) 5.1NiO/Z_m_; (**c**) 1.8NiO/Z_m_; (**d**) 4.8NiO/Z_hy_; (**e**) 1.7NiO/Z_hy_. α, β and γ: temperature ranges for the reduction of NiO species interacting differently with the support.

The reduction profile of the 5.1NiO/Z_m_ sample (Figure 4b) showed an intense H_2_ consumption peak in the 573–673 K range with a maximum at 640 K, close in position to that of unsupported NiO (Figure 4a), and a very weak H_2_ consumption spread up to 923 K. The 1.8NiO/Z_m_, 4.8NiO/Z_hy_ and 1.7NiO/Z_hy_ samples (Figure 4, profiles c–e) showed a very similar reduction profile with an intense and narrow peak at about 700 K and a broad hydrogen consumption in the 750–923 K range, with the integrated intensity of the peaks being proportional to the nickel content. Several authors have suggested that for NiO supported on ZrO_2_ or modified ZrO_2_, the stronger the interaction, the higher the reduction temperature [32,33,48,49,50,51]. Accordingly, the intense peak with a maximum at 640 K was attributed to NiO species not or weakly interacting with the ZrO_2_ surface, named α species; the peak and the envelope in the range of 673-873 K were ascribed to species interacting with the support, named β and γ, with the γ species being more strongly interacting than the β species are [51]. The similarity in shape and position of the reduction profiles of 1.8NiO/Z_m_, 4.8NiO/Z_hy_ and 1.7NiO/Z_hy_ precursors indicated that the strength of the NiO interaction with the support did not depend on the specific starting material adopted for the impregnation, Z_m_ or Z_hy_. As shown in Table 3, for these samples, the H_2_ consumption was only due to β and γ NiO species and was much higher than that due to β and γ species in the 5.1NiO/Z_m_ sample (100% vs 11%). The lower β and γ species amounts in 5.1NiO/Z_m_ than in 1.8NiO/Z_m_ precursors (Figure 4, profiles b and c, respectively) indicated that the amount of NiO interacting with the monoclinic ZrO_2_ decreased with nickel content, due to saturation of the low number of surface sites available on Z_m_ to anchor Ni^2+^ species.

The characterization results for NiO/ZrO_2_ catalyst precursors indicated that the use of amorphous Z_hy_ as a starting material for the preparation of samples favoured the spreading of NiO on the support (FESEM and Raman evidence), particularly at high Ni contents. The higher number of sites available on Z_hy_ than on Z_m_ to anchor Ni^2+^ species during the impregnation step allowed the formation of smaller NiO particles on Z_hy_ than on Z_m_ during the subsequent thermal treatment, implying a higher number of Ni sites strongly interacting with Z_hy_ than with Z_m_ (TPR evidence).

#### 3.1.3. Ni/ZrO_2_ Catalysts

**Figure 5:**
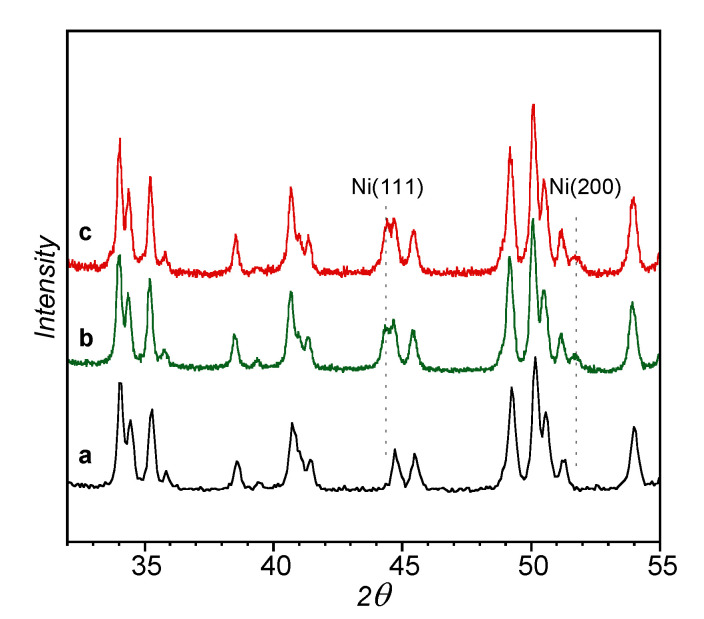
XRD patterns of monoclinic ZrO_2_ and activated Ni/ZrO_2_ catalysts. (**a**) Z_m_; (**b**) 4.8Ni/Z_hy_ (*red*); (**c**) 4.8Ni/Z_hy_ (*ox-red*).

XRD patterns of Ni/ZrO_2_ catalysts, irrespective of the different activation treatments, showed reflections assigned to the (111) and (200) planes of Ni metal (JCPDS card 04-0850) and to monoclinic ZrO_2_ (JCPDS card 37-1484), as shown in Figure 5 for both 4.8Ni/Z_hy_ *ox-red* and *red*, as an example.

Ni crystallite sizes, obtained by the Scherrer equation [41] using the (200) and/or (111) reflections, ranged from 18 to 35 nm for all of the samples (Table 1).

**Figure 6:**
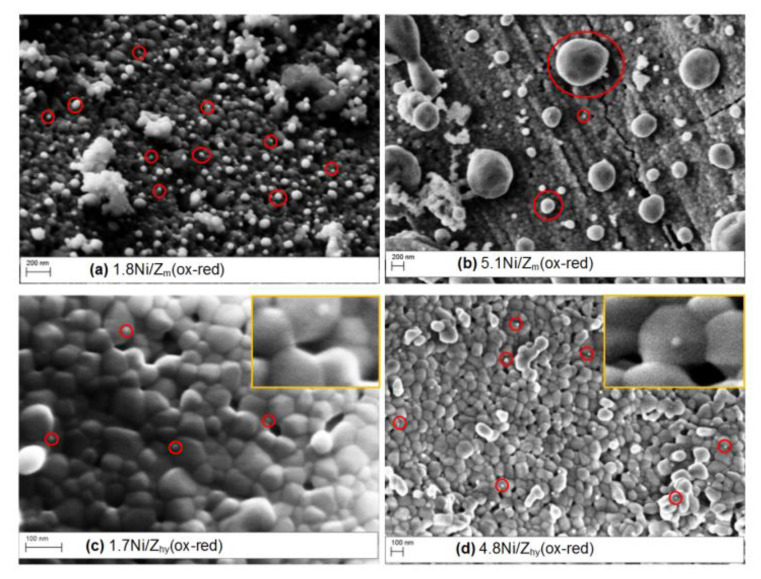
FESEM images of Ni/Z_hy_ (*ox-red*) and Ni/Z_m_ (*ox-red*) catalysts. Red circles and magnifications in sections (**c**) and (**d**) highlight some Ni particles.

FESEM micrographs showed Ni particles larger in size with respect to XRD, indicating that, as for NiO, Ni crystallite aggregates were present on the zirconia surface. For Ni/Z_m_ catalysts, the Ni particle sizes (Table 2) were in the range of 5–75 nm in the 1.8Ni/Z_m_ (*ox-red*) sample (Figure 6a), and in the range of 30–1000 nm (highly heterogeneous in size) in the 5.1Ni/Z_m_ (*ox-red*) sample (Figure 6b). For Ni/Z_hy_ catalysts, small Ni particles of about 5–45 nm in diameter, in both the 1.7Ni/Z_hy_ (*ox-red*) and 4.8Ni/Z_hy_ (*ox-red*) samples, were detected (Figure 6c,d). Particle size values in the same ranges were found for the corresponding Ni/Z_hy_ (*red*) and Ni/Z_m_ (*red*) catalysts (Table 2).

Both the *ox-red* and *red* activation treatments caused a small particle sintering during the reductive process. In fact, instead of the expected decrease in particle size due to oxygen removal (based on NiO and Ni volume atomic densities, ρ_NiO_ = 2.69·10^22^ Ni-atoms/cm^3^ and ρ_Ni_ = 9.13·10^22^ Ni-atoms/cm^3^, respectively) [45], we observed a small increase in size (compare d_FESEM_ of Ni with d_FESEM_ of NiO particles, Table 2). This result suggests that the NiO-ZrO_2_ interaction in the catalyst precursors was strong enough to limit the aggregation of Ni particles during the reduction process. In agreement, metal particle sizes of the unsupported Ni metal sample ranged from 40 to 1500 nm, much larger than those detected on Ni/ZrO_2_ samples (Table 2).

Overall, irrespective of the activation treatments, *ox-red* or *red*, Ni particles were smaller and more homogeneous in size in Ni/Z_hy_ than in Ni/Z_m_ catalysts, particularly at high Ni contents.

#### 3.1.4. Dispersion of Ni in the Ni/ZrO_2_ Catalysts

To interpret the catalytic results (see below), the determination of Ni dispersion values is an important step in shedding light on the nature of active sites. The most frequently used method to evaluate the dispersion of the active phase is selective hydrogen chemisorption that allows the metal surface area measuring the H_2_ uptake to be determined. However, even in the samples with high Ni content and small particle size (as revealed by FESEM), the expected H_2_ uptake is too low to be confidently detected with our laboratory facilities (TPD and/or H_2_ chemisorption). As an alternative, the Ni dispersion could be evaluated by a physical technique able to measure particle diameters, d_i_. Among the electron microscopy techniques, TEM is considered the most reliable and accurate physical technique for d_i_ measurements due to a resolution as high as 0.1 nm, whereas FESEM microscopy has a lower sensitivity limit (5 nm). As the particle sizes in our samples (Table 2) were above 5 nm in size, we confidently used FESEM microscopy to measure d_i_ values.

**Figure 7:**
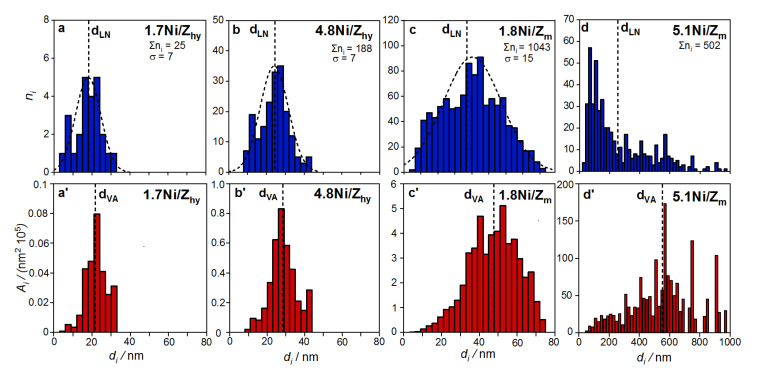
Ni-particle number (n_i_) (**a**–**d**) and Ni-particle surface (A_i_ = πn_i_d_i_^2^) (**a’**–**d’**) distributions as a function of Ni-particle diameters (d_i_) for 1.7Ni/Z_hy_ (**a**,**a’**), 4.8Ni/Z_hy_ (**b**,**b’**), 1.8Ni/Z_m_ (**c**,**c’**) and 5.1Ni/Z_m_ (**d**,**d’**) samples. Dotted lines identify the length-number mean diameter (d_LN_) or volume-area mean diameter (d_VA_) values; Σn_i_ stands for the total number of Ni particles from FESEM images. For samples showing a normal distribution curve of Ni particles size, the curve and the corresponding standard deviation σ are also reported.

From FESEM image processing, we obtained a distribution of Ni-particle size, d_i_, which was nearly symmetrical for the Ni/Z_hy_ samples and the 1.8Ni/Z_m_ sample (Figure 7a–c), whereas it was asymmetrical and spread over a broad range of d_i_ sizes (up to 1000 nm) for the 5.1Ni/Z_m_ sample (Figure 7d). From these distributions, the length-number mean diameter, d_LN_, could be calculated (Table 2), as d_LN_ = Σd_i_/Σn_i_ [45], where n_i_ is the number of metal particles with a specific diameter d_i_. As reported in the literature [45,52], for phenomena depending on the particle surface, such as the catalytic activity, the mean diameter value that gives a better account of the total metal surface area is the volume/area mean diameter, d_VA_ (d_VA_ = Σ_i_n_i_d_i_^3^/Σn_i_d_i_^2^ = 6·Σ_i_n_i_V_i_/Σn_i_A_i_), which is directly related to the metal surface area [45]. In fact, in a particle-size distribution, large particles give the major contribution to the total metal surface area, as shown in the particle-surface distribution of Ni/ZrO_2_ samples at increasing particle size (Figure 7a’–d’). Therefore, the dispersion was calculated from the d_VA_ values, which are always higher than the d_LN_ values [53]. The difference between d_VA_ and d_LN_ increased as the distribution broadened towards larger d_i_ sizes. In agreement, for the 1.7Ni/Z_hy_, 1.8Ni/Z_m_ and 4.8Ni/Z_hy_ samples, d_VA_ values were similar to the d_LN_ values, whereas for the 5.1Ni/Z_m_ sample, the d_VA_ value was about twice that of the d_LN_ value (Figure 7 and Table 2). FESEM image processing gave similar Ni dispersion values for the 1.7Ni/Z_hy_ and 4.8Ni/Z_hy_ samples (about 4%), slightly lower for the 1.8Ni/Z_m_ sample (about 2%), and about 20 times lower for the 5.1Ni/Z_m_ sample (Table 2).

#### 3.1.5. Ni/ZrO_2_ Used Catalysts

XRD patterns of all the used catalysts were similar to those of activated catalysts, showing the presence of both monoclinic ZrO_2_ and Ni metal phases. The Ni mean crystallite size, estimated by XRD (Table 1), remained nearly unchanged under catalytic conditions.

**Figure 8:**
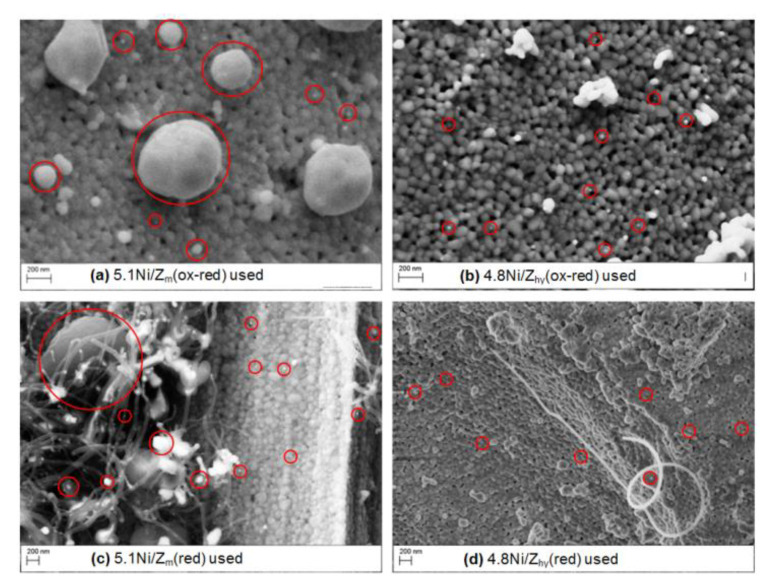
(**a**–**d**) FESEM images of 4.8Ni/Z_hy_ and 5.1Ni/Z_m_ used catalysts. Red circles highlight some Ni particles.

FESEM analysis confirmed that Ni particles remained unchanged both in size and distribution after catalytic runs (Table 2), as revealed by comparing Figure 6b,d with Figure 8a,b for high-loaded catalysts as an example. These results supported the idea that, irrespective of dispersion, Ni metal particles did not undergo sintering under catalytic conditions. Carbon whiskers encapsulating Ni particles were detected on both 5.1Ni/Z_m_ (*red*) and 4.8Ni/Z_hy_ (*red*) used catalysts, whereas they were not observed on the Ni/Z (*ox-red*) used catalysts (Figure 8). This suggests that Ni particles were firmly anchored to the ZrO_2_ support in Ni/Z (*ox-red*) catalysts, whereas in Ni/Z (*red*) catalysts, some Ni species showed a different reactivity that led to the growth of C whiskers incorporating Ni.

**Figure 9:**
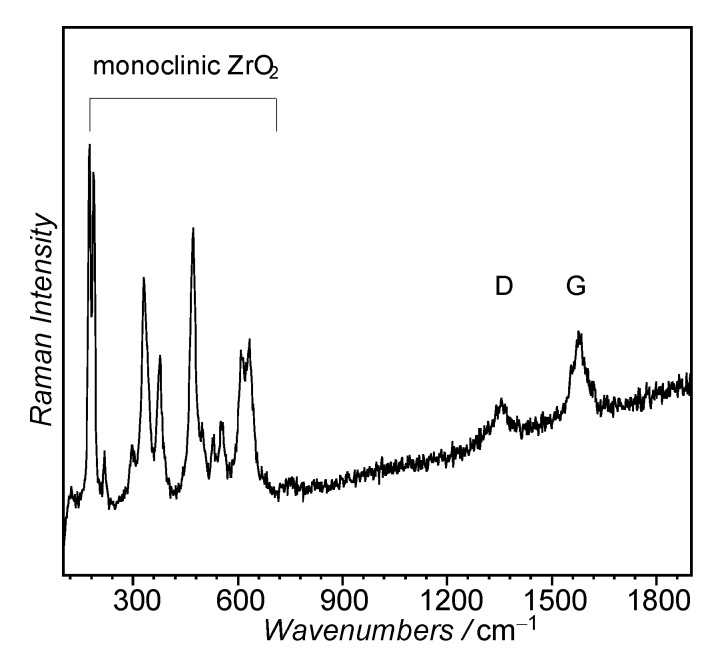
Raman spectrum of the 5.1Ni/Z_m_ (*red*) used catalyst. D and G identify disordered-graphite and graphite bands, respectively.

Raman spectroscopy, in agreement with FESEM results, clearly identified carbonaceous deposits on both 5.1Ni/Z_m_ (*red*) and 4.8Ni/Z_hy_ (*red*) used catalysts. As illustrated in Figure 9 for the 5.1Ni/Z_m_ (*red*) used sample as an example, in addition to the bands due to monoclinic ZrO_2_, bands at about 1320 cm^−1^ (D band) and 1584 cm^−1^ (G band) were recorded. The G band (‘graphite peak,’ E_2g_ symmetry) is characteristic of a perfect graphite lattice, whereas the D band (‘defect peak,’ A_1g_ symmetry) is related to a defective graphitic lattice [53]. The nonoverlapping of the D and G bands is consistent with the presence of a structured carbon; the band at about 1584 cm^−1^ could originate from graphitic carbon types on the catalyst’s surface and that at about 1320 cm^−1^ could originate from carbon nanoparticles or defective filamentous carbon. The relative intensity of the D band to the G band, I_D_/I_G_, has been taken as a measure of the structural disorder of the carbon material, a high ratio indicating a large amount of disorder in the structure and more reactive carbon species [24]. An I_D_/I_G_ value of 0.9 was obtained by the curve fitting procedure for the 5.1Ni/Z_m_ (*red*) used sample, indicating the presence of a significant contribution of graphite-type structures.

Overall, characterization results of catalysts, before and after catalytic runs, showed that the starting material adopted for the preparation, Z_hy_ or Z_m_, (i) affected the Ni particle sizes (smaller for the high-surface Z_hy_) because it affected the NiO particle sizes in the catalyst precursors; (ii) it did not affect the strength of the interaction between Ni particles and the ZrO_2_ surface. In all the catalysts, the activation treatment of the NiO/ZrO_2_ precursors did not affect the Ni particle sizes or the Ni-ZrO_2_ interactions that were strong enough to prevent the sintering of the Ni metal particles.

### 3.2. Catalytic Activity

**Figure 10:**
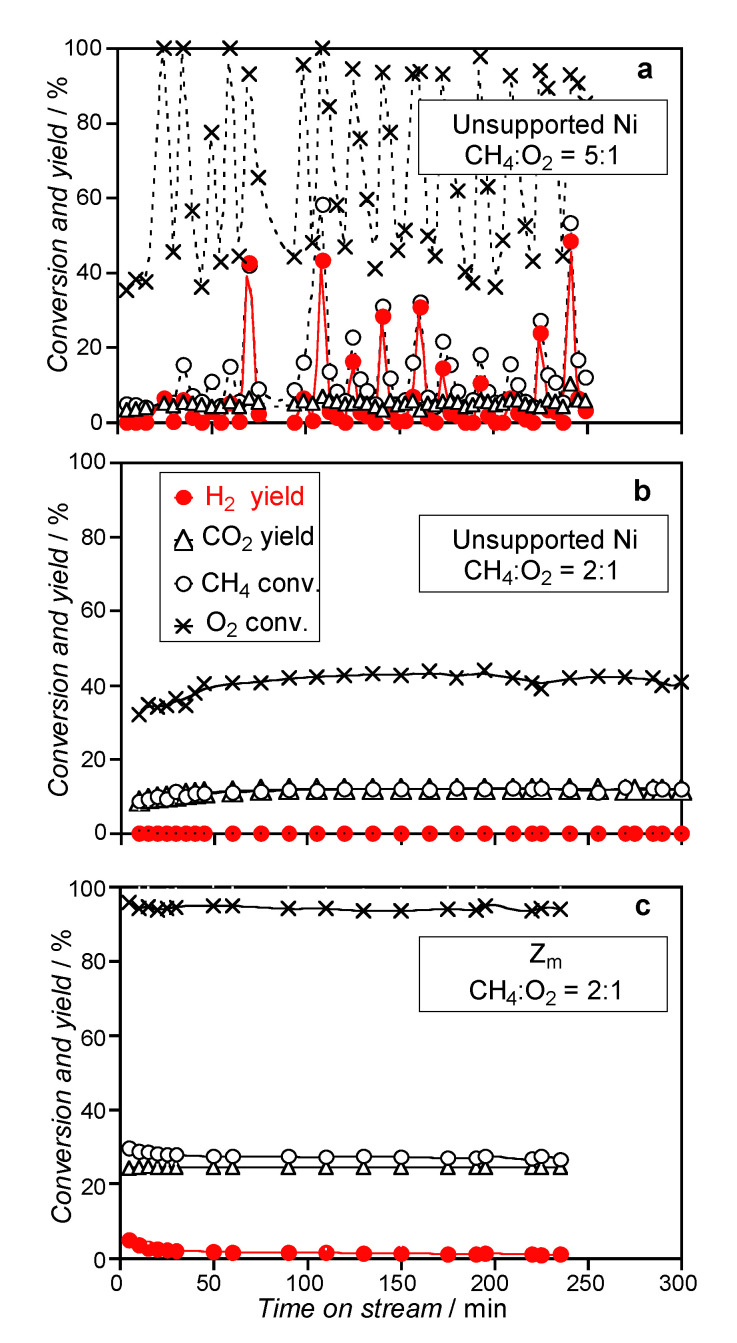
Catalytic activity of unsupported Ni metal and monoclinic ZrO_2_ (Z_m_) with reactant mixtures at different CH_4_:O_2_ ratios. CH_4_ and O_2_ conversions and H_2_ and CO_2_ yields as a function of time on stream at 1023 K for *ox-red*-activated catalysts. (**a**): [CH_4_] = 2%, [O_2_] = 0.4%. (**b**,**c**): [CH_4_] = 2%, [O_2_] = 1%, N_2_ as balance; total flow rate = 150 cm^3^ (STP)·min^−1^.

Unsupported Ni as foil or wires was reported to be somewhat active for the CH_4_-CPO using a methane-rich mixture CH_4_:O_2_ = 5:1, with a peculiar oscillatory activity [54,55]. Unsupported Ni metal powder was tested for the CH_4_-CPO using the methane-rich mixture and the stoichiometric mixture (CH_4_:O_2_ = 2:1). Irrespective of the activation procedure (*ox-red* or *red*), Ni metal powder showed an oscillatory on–off activity at 1023 K with the methane-rich mixture (Figure 10a), whereas it was inactive for syngas production with the stoichiometric mixture, showing only activity for the CH_4_ total combustion (Figure 10b).

The Z_m_ support was inactive for the CH_4_-CPO in the temperature range of 773–1023 K with the CH_4_:O_2_ = 2:1 mixture and, irrespective of the activation treatment, yielded a total oxidation of CH_4_ above 823 K (maximum CO_2_ yield of about 25% at 1023 K, Figure 10c).

Unlike ZrO_2_ and unsupported Ni metal, Ni/ZrO_2_ catalysts were active for the CH_4_-CPO, indicating that the metal–support interaction plays a key role in developing Ni active species. However, the catalytic behaviour markedly depended on the activation procedure (*ox-red* or *red*), as illustrated in the following sections.

#### 3.2.1. Catalysts Activated by Oxidation–Reduction Treatment

**Figure 11:**
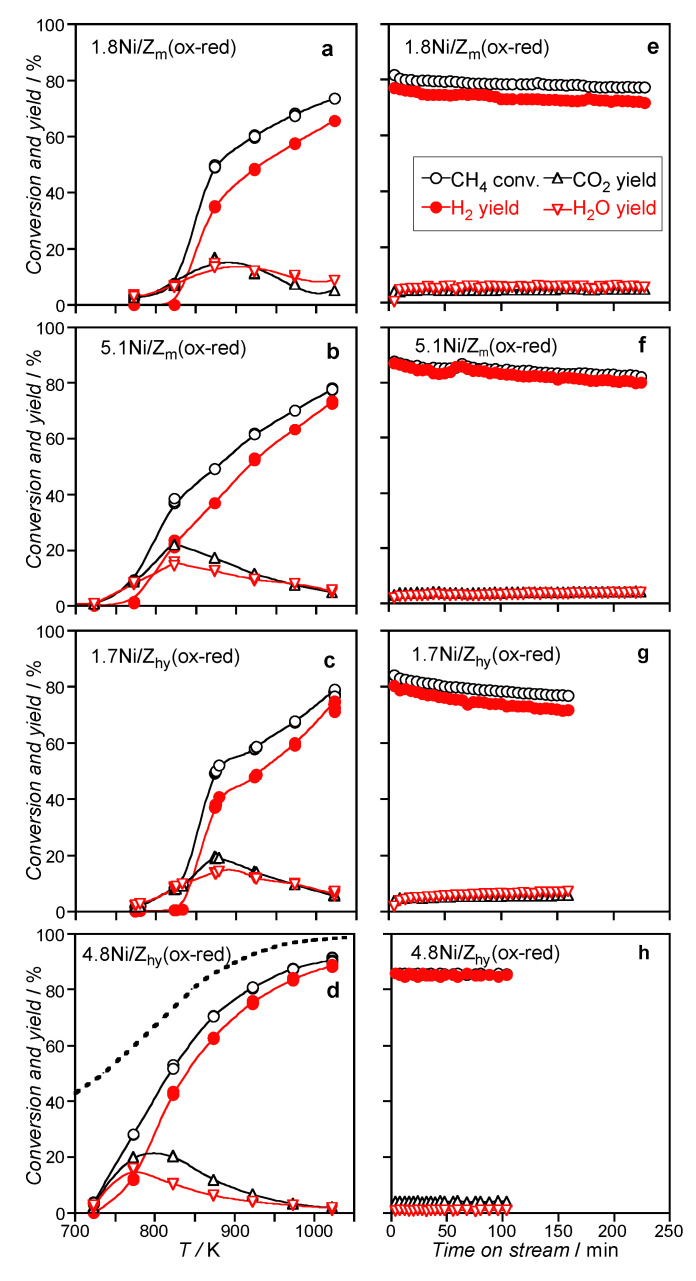
Catalytic activity (CH_4_ conversion and H_2_, CO_2_ and H_2_O yields) of Ni/ZrO_2_ catalysts after *ox-red* activation treatment in experiments as a function of temperature (left side: (**a**–**d**)) or as a function of time on stream at 1023 K (right side: (**e**–**h**)). Reactant mixture: [CH_4_] = 2%, [O_2_] = 1%, N_2_ as balance; total flow rate = 150 cm^3^ (STP)·min^−1^. Dotted line: CH_4_ conversion for the same mixture calculated at thermodynamic equilibrium (data from Ref. [11]).

**Figure 12:**
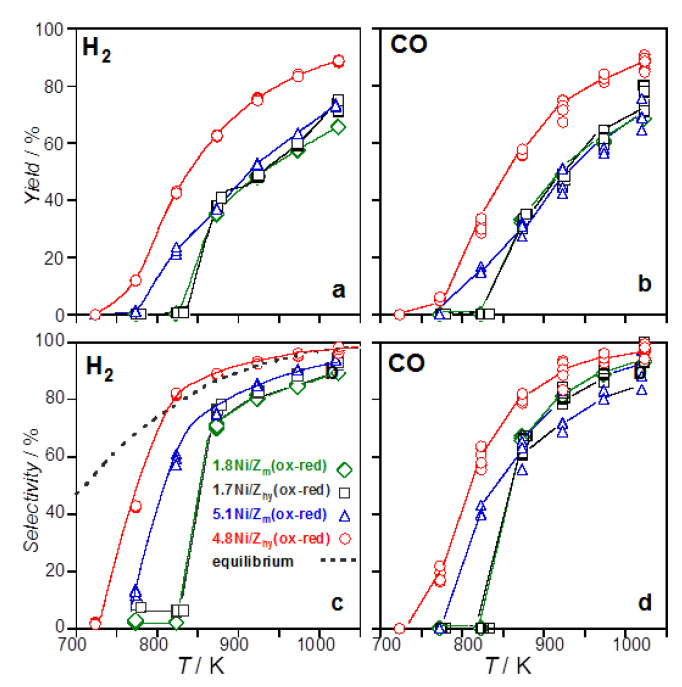
Comparison of activity and selectivity for Ni/ZrO_2_ catalysts after *ox-red* activation treatment. H_2_ yield and selectivity (**a**,**c**) and CO yield and selectivity (**b**,**d**) as a function of temperature. Reactant mixture: [CH_4_] = 2%, [O_2_] = 1%, N_2_ as balance; total flow rate = 150 cm^3^ (STP)·min^−1^. Dotted line: H_2_ selectivity for the same mixture calculated at thermodynamic equilibrium (data from Ref. [11]).

All the Ni/Z_m_ (*ox-red*) and Ni/Z_hy_ (*ox-red*) catalysts were highly active (Figure 11a–d and Figure 12a,b) and selective (Figure 12c,d) for the CH_4_-CPO at 1023 K with the CH_4_:O_2_ = 2:1 mixture. The most active 4.8Ni/Z_hy_ (*ox-red*) sample reached, at 1023 K, a CH_4_ conversion and H_2_ selectivity close to the equilibrium (90% and 95%, respectively [14], Figure 11d and c).

For all the catalysts, decreasing the temperature below 1023 K decreased the H_2_ selectivity (H_2_ yield lower than CH_4_ conversion), indicating the occurrence of side reactions. The formation of similar amounts of CO_2_ and H_2_O as by-products suggested the occurrence of CH_4_ total combustion, with a volcano-shape activity trend as a function of temperature (maximum CO_2_ yield of 20%). Below 923 K, more evident for the concentrated 5.1Ni/Z_m_ (*ox-red*) and 4.8Ni/Z_hy_ (*ox-red*) samples, the H_2_O yield was slightly lower than the CO_2_ yield, and the H_2_ yield was slightly higher than the CO yield, suggesting that the WGS reaction occurred to a small extent. For all the catalysts, the activity and selectivity for the CH_4_-CPO dropped to zero, further lowering the temperature, and they were completely restored by a subsequent temperature increase, indicating that side-reactions causing irreversible deactivation did not occur.

The activity and selectivity as a function of time on stream at a set temperature were stable for the 4.8Ni/Z_hy_ (*ox-red*) catalyst, but were slightly decreased for all the other catalysts (Figure 11e–h). The reproducibility of activity and selectivity in the whole temperature range was observed in each run and in consecutive runs (Figure S3a), with the error affecting the conversion values of, at maximum, 7%. All of these results suggest that no significant changes in the amount and/or type of active Ni sites occurred during the reaction and that the slight decrease in activity with time possibly arose from the deposition of a very small amount of coke on Ni active sites, in agreement with the literature [23].

Similar H_2_ and CO yields (Figure 12a,b) and selectivities (Figure 12c,d) were observed for dilute 1.8Ni/Z_m_ (*ox-red*) and 1.7Ni/Z_hy_ (*ox-red*) catalysts, indicating that at low Ni loading, the starting supports allowed the formation of similar Ni active sites. The increase in Ni loading caused a different behaviour of catalysts prepared using Z_m_ or Z_hy_ starting materials. Specifically, for Ni/Z_m_ (*ox-red*) catalysts, the increase in Ni loading lowered the reaction light-off of about 50 K, leaving the H_2_ and CO yield and selectivity values unchanged above the light-off temperature. By contrast, for Ni/Z_hy_ (*ox-red*) catalysts, the increase in Ni loading caused a more consistent lowering of the reaction light-off temperature (about 100 K), giving higher H_2_ and CO yield and selectivity values for 4.8Ni/Z_hy_ (*ox-red*) than for 1.7Ni/Z_hy_ (*ox-red*) catalysts (Figure 12a–d). Therefore, the 4.8Ni/Z_hy_ (*ox-red*) catalyst showed the best performances, exhibiting the lowest CPO light-off temperature and the highest H_2_ and CO yield and selectivity values.

As 1.8Ni/Z_m_ (*ox-red*) and 1.7Ni/Z_hy_ (*ox-red*) catalysts showed similar activity, the starting material Z_hy_ or Z_m_ used for the impregnation seemed to have no effect on the active site reactivity. The evidence that the 4.8Ni/Z_hy_ (*ox-red*) catalyst with the smallest Ni particles had an activity higher than that of the 5.1Ni/Z_m_ (*ox-red*) catalyst with the largest particles (FESEM evidence, Table 2) might raise the expectation that Ni dispersion favours catalytic activity.

To verify whether dispersion can be the key parameter affecting activity, the dependence of the rate of H_2_ production (R_H2_/molecules s^−1^·g^−1^) on the number of exposed Ni atoms, N_Ni(exp)_ (Ni(exp) atoms·g^−1^), was analysed for catalysts with different Ni dispersions (Table 2). The N_Ni(exp)_ value of the 4.8Ni/Z_hy_ (*ox-red*) catalyst (1.8·10^19^ atoms·g^−1^) was about 20 times higher than that of the 5.1Ni/Z_m_ (*ox-red*) catalyst (9.4·10^17^ atoms·g^−1^), whereas the rate R_H2_ at 823 K for the 4.8Ni/Z_hy_ (*ox-red*) catalyst was only about twice as high than that for the 5.1Ni/Z_m_ (*ox-red*) catalyst (2.2·10^19^ vs. 1.2·10^19^ molecules·s^−1^·g^−1^, respectively). The lack of a linear correlation between R_H2_ and N_Ni(exp)_ indicated that neither all nor a constant fraction of exposed Ni atoms were the active sites, only Ni species with peculiar properties. As a consequence, neither the different Z_hy_ or Z_m_ starting material alone nor Ni dispersion alone was the factor affecting the activity of Ni sites for CH_4_-CPO.

#### 3.2.2. Catalysts Activated by Reduction Treatment

**Figure 13:**
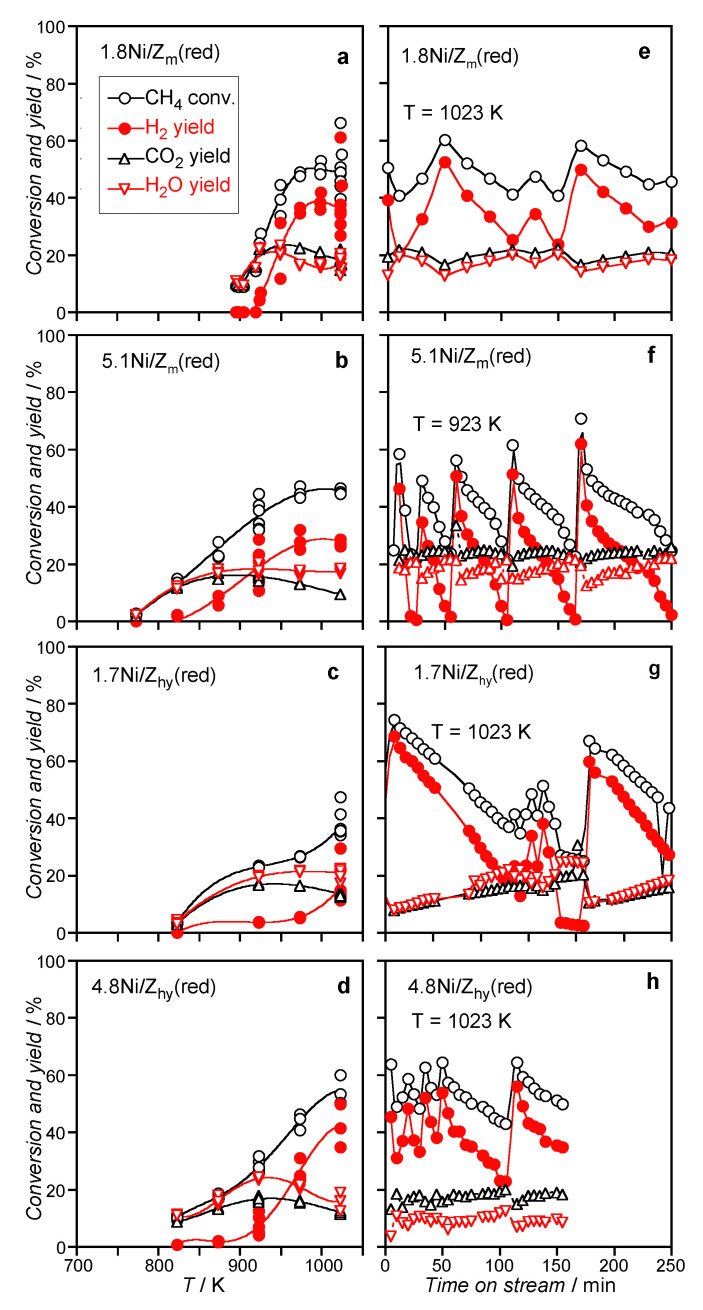
Catalytic activity (CH_4_ conversion and H_2_, CO_2_ and H_2_O yields) of Ni/ZrO_2_ catalysts after *red* activation treatment in experiments as a function of temperature (left side: **a**–**d**) or as a function of time on stream at set temperatures (right side: **e**–**h**). Reactant mixture: [CH_4_] = 2%, [O_2_] = 1%, N_2_ as balance; total flow rate = 150 cm^3^ (STP)·min^−1^.

All the Ni/Z_m_ (*red*) and Ni/Z_hy_ (*red*) catalysts were less active (Figure 13a–d) and selective (Figure S4a,b) for the CH_4_-CPO than the corresponding *ox-red* samples in the whole temperature range. They gave scattered values of H_2_ and CO yields and no scattered combustion by-product values. Both the scattered H_2_ and CO yields and the lower activity of *red* samples with respect to the *ox-red* samples suggested that the *red* activation treatment generated active sites in a lower amount and with features unlike those obtained by the *ox-red* procedure.

For all Ni/Z_m_ (*red*) and Ni/Z_hy_ (*red*) catalysts, the scattered trend of activity was reproduced in subsequent runs (Figure S3b), indicating that, as for Ni/Z_m_ (*ox-red*) and Ni/Z_hy_ (*ox-red*) catalysts, no irreversible modification of the active Ni sites occurred during catalytic runs.

By investigating the activity at a set temperature, the Ni/Z_m_ (*red*) and Ni/Z_hy_ (*red*) catalysts exhibited CH_4_ conversion and H_2_ and CO yields markedly changing as a function of time on stream with *in-phase saw-tooth shape* oscillations (Figure 13e–h), indicating an oscillating behaviour of the CH_4_-CPO reaction. In the oscillating cycles, the total oxidation side-reaction occurred for all the catalysts, as evidenced by the CH_4_ conversion always being higher than the H_2_ yield and H_2_O and CO_2_ formation.

WGS side reactions also occurred to a small extent, as the H_2_ yield was slightly higher than that of CO, and the CO_2_ yield was slightly higher than that of H_2_O. The slightly oscillating behaviour of CO_2_ and H_2_O yields (Figure 13e–h), opposite in phase to that of the CH_4_-CPO, suggested an oscillating behaviour even for the WGS reaction. The oscillation of the WGS reaction might arise from the significant change in CO concentration as the reactant, consequent to the CH_4_-CPO oscillation. As the change in the relative amount of CO_2_ and H_2_O yields was small and arose from WGS oscillation, the amounts of CO_2_ and H_2_O arising from CH_4_ combustion were constant, indicating that the CH_4_ total oxidation did not oscillate. In addition, as the CO_2_ yield in the Ni/Z (*red*) catalysts was roughly similar to that observed for the Z_m_ support (see Figure 10c), we suggest that the active sites yielding CH_4_ total oxidation are support sites, and those yielding oscillating CH_4_-CPO and WGS behaviours are Ni sites.

The remarkable oscillation of the CH_4_-CPO reaction for the Ni/Z_m_ (*red*) and Ni/Z_hy_ (*red*) catalysts can be tentatively explained according to evidence widely discussed in the literature for various metal or supported metal systems [55,56,57,58,59,60,61,62]. For supported noble metal catalysts (Ru and Pd), the oscillating behaviour was ascribed to a periodical oxidation–reduction of the active metal sites, with the metal species being active for the CH_4_-CPO and the oxidised species being active for the CH_4_ combustion [58,61]. In particular, an oxidation/reduction front of Pd nanoparticles was suggested to move through the catalytic fixed-bed, yielding the periodical predominance of the CH_4_ total oxidation with respect to the partial oxidation [61]. An *operando* XRD study on a Ni foil showed unambiguously that the oscillations of the CH_4_-CPO reaction were due to the reversible oxidation of Ni metal to NiO [63]. For Ni and Co foils [60] and Ni wires [56], it was suggested that the reversible oxidation–reduction of surface sites depended on the gas-phase composition and on changes in the local temperature due to the exothermicity of the reactions at the metal surface.

In agreement with all these findings, we suggest that in both Ni/Z_m_ (*red*) and Ni/Z_hy_ (*red*) catalysts, the CH_4_-CPO oscillating behaviour arose from the periodical oxidation–reduction of some Ni sites, which were CPO-inactive when oxidised and CPO-active when reduced. Unlike supported Ru and Pd catalysts [58,61], the oxidised Ni species that formed during the oscillation cycle contributed little to the CH_4_ total oxidation, as in our catalysts, CH_4_ total oxidation did not oscillate.

**Figure 14:**
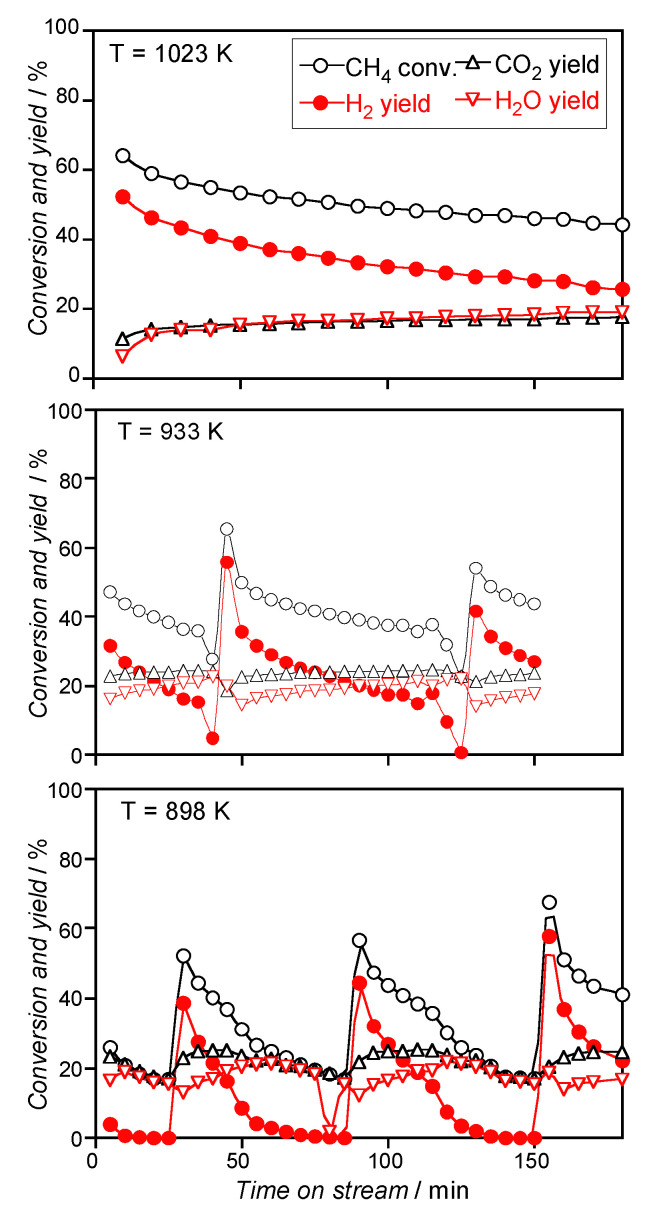
CH_4_ conversion and H_2_, CO_2_ and H_2_O yields on 5.1Ni/Z_m_ (*red*) catalyst as a function of time on stream for specific temperatures. Reactant mixture: [CH_4_] = 2%, [O_2_] = 1%, N_2_ as balance; total flow rate = 150 cm^3^ (STP)·min^−1^.

The oscillating behaviour depended on temperature, contact time and O_2_ content in the mixture. Specifically, (i) the trends of conversions and yields changed form or disappeared, depending on temperature (Figure 14), as the surface reactions causing changes in the Ni oxidation state depended on temperature by different activation energy values; (ii) oscillations disappeared, increasing the total flow rate (Figure S5), in agreement with the literature, showing that short contact times favoured CH_4_-CPO compared to total oxidation [63]; (iii) oscillations appeared when the O_2_ content was above 0.5% (Figure S6), as a higher oxygen coverage of the metal surface favoured the oxidation of Ni sites [60].

To explain why Ni/Z_m_ (*red*) and Ni/Z_hy_ (*red*) catalysts yielded oscillations, whereas Ni/Z_m_ (*ox-red*) and Ni/Z_hy_ (*ox-red*) did not, we rationalized our results considering carbon balance, oscillation reproducibility, and Ni particle size for the used samples. As, during the oscillation cycles, the carbon balance was satisfactory, the oscillating behaviour could not be related to the process of the covering/restoring of active Ni sites (carbon deposition followed by auto-thermally induced carbon oxidation). In addition, as the oscillating behaviour was the same in subsequent runs, the number and features of Ni sites causing oscillations were not affected by aging in the CH_4_-CPO feed and by the re-activation treatment between two consecutive runs. Recalling that for all the catalysts, the *red* activation treatment yielded Ni metal particles similar in size to those obtained by the *ox-red* activation treatment (see Table 2), the oscillating activity of catalysts could not be attributed to Ni particles of specific size. Therefore, we suggest that the *red* activation treatment yielded specific Ni sites able to easily change their oxidation state, inducing an oscillating process.

### 3.3. Surface Species Evolution during Activation Treatments by in situ FTIR

To gain an insight into the Ni surface species that might be involved in the oscillating activity of Ni/Z_m_ (*red*) and Ni/Z_hy_ (*red*) catalysts, in situ FTIR analysis was performed in a gas flow simulating the activation treatments of the catalyst precursors.

**Figure 15:**
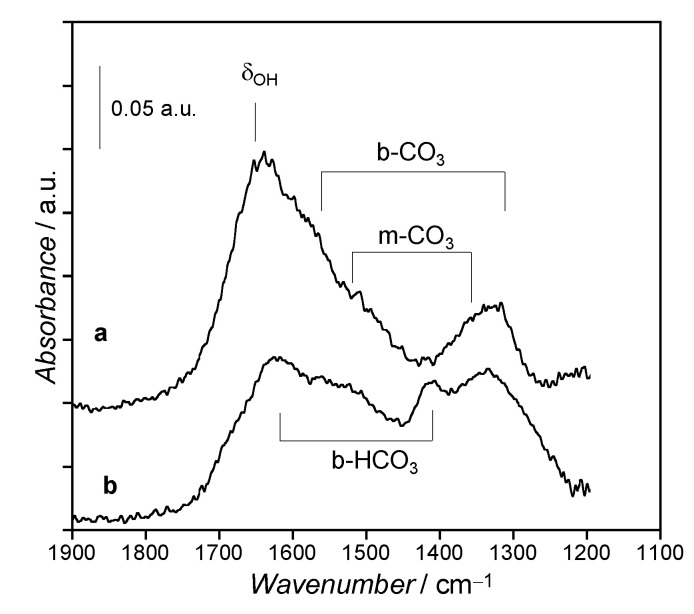
FTIR spectra of surface species recorded in air at 298 K. (**a**) Z_m_ support; (**b**) 4.8NiO/Z_hy_ catalyst precursor.

The spectra of the Z_m_ support and the 4.8NiO/Z_hy_ precursor recorded in air at 298 K (Figure 15) showed a broad band at about 3600 cm^−1^ due to H-bonded OH (ν_OH_, spectral region not shown) and an envelope in the 1650–1250 cm^−1^ region, containing bands characteristic of adsorbed water (at about 1630 cm^−1^, δ_OH_ mode), monodentate carbonates (m-CO_3_, at 1475 and 1355 cm^−1^), bidentate carbonates (b-CO_3_, at 1560 and 1330 cm^−1^) and bidentate bicarbonates (b-HCO_3_, at 1690 and 1410 cm^−1^) adsorbed on the zirconia surface [64,65,66,67,68].

**Figure 16:**
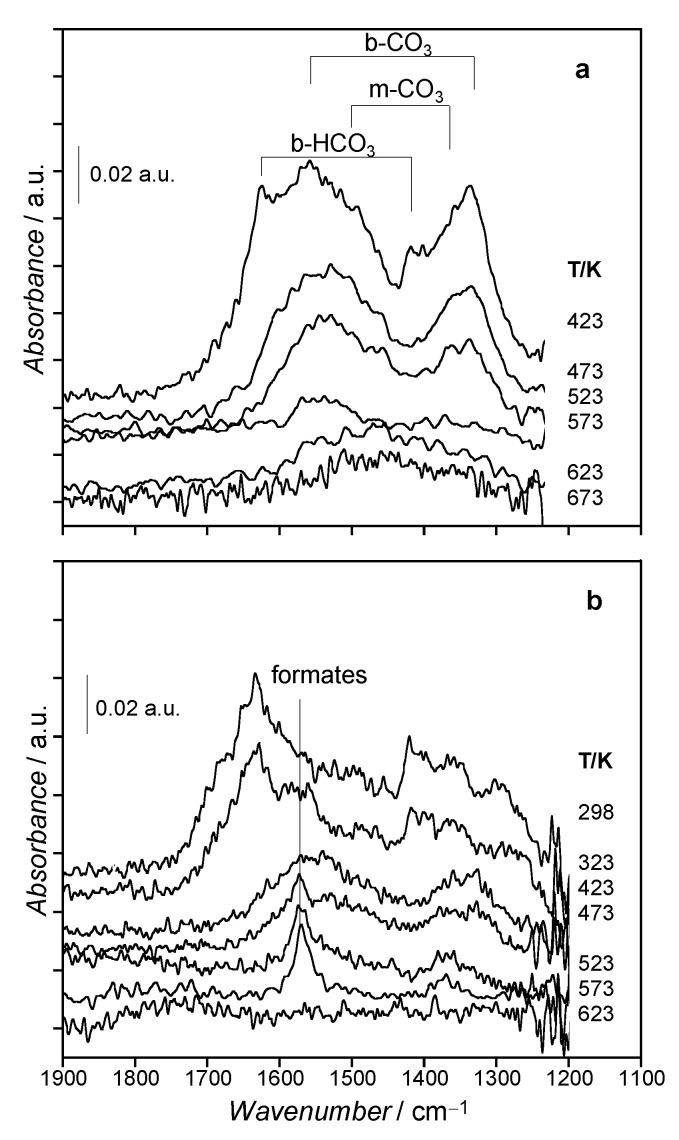
In situ FTIR spectra of surface species during activation treatments on 4.8NiO/Z_hy_ catalyst precursor. Spectra recorded in O_2_/N_2_ oxidative flow (**a**), and in H_2_/N_2_ reductive flow (**b**) at increasing temperature.

The exposure of the 4.8NiO/Z_hy_ precursor to an O_2_/N_2_ flow at increasing temperature, simulating the oxidative step of the *ox-red* activation procedure, produced a progressive decrease in intensity of the bands due to adsorbed H_2_O, carbonates and bicarbonates, until their disappearance above 573 K (Figure 16a).

The exposure of the 4.8NiO/Z_hy_ precursor to an H_2_/N_2_ flow, at increasing temperature, simulating the *red* activation procedure, produced a progressive decrease in intensity of adsorbed H_2_O and carbonates/bicarbonates bands. At 473 K, a new band at about 1575 cm^−1^ appeared, whose intensity reached a maximum at 573 K and then decreased with temperature. All the bands disappeared above 623 K (Figure 16b). The band at 1575 cm^−1^ was assigned to formate species [69], arising from the reduction of the adsorbed carbonates/bicarbonates surface species in the H_2_ flow.

The formation of reduced carbon-containing species solely after the *red* activation treatment of the precursors could account for the dependence of the catalytic behaviour on the activation procedures. During the *red* activation, carbonates species adsorbed on the support could be reduced to formates that, at higher temperature, are supposed to form carbide-like species (C^δ−^), not detectable by IR. The C^δ−^ species located on the ZrO_2_ surface in proximity of the Ni particles could induce a positive charge on Ni atoms at the boundary between metal particles and the support, yielding Ni^δ+^ surface species. These species could easily change their oxidation state, thus favouring the oscillating behaviour of the CH_4_-CPO reaction. As the oscillating catalytic behaviour was retained by subsequent *red-ox* cycles, it can be assumed that the C^δ−^ species, once formed, are stable on the surface of *red*-activated catalysts. The formation of carbide-like species in Ni/Z_m_ (*red*) and Ni/Z_hy_ (*red*) catalysts, hypothesized based on FTIR results, can find some correspondence in the presence of carbon nanotubes observed by FESEM. In fact, by consensus, the presence of nickel carbide species was well recognised to be involved in the induction step of the carbon nanotubes formation on Ni metal particles [70]. Conversely, when the catalysts precursors were activated by the *ox-red* procedure, adsorbed carbonates desorbed during the oxidation step, and carbide-like species could not form during the reduction step. Consequently, Ni active sites with a pure metal character formed, thus preventing the oscillating phenomenon.

### 3.4. Hypothesis on Active Sites for the CH_4_-CPO

The activity of both Ni/Z_m_ (*ox-red*) and Ni/Z_hy_ (*ox-red*) catalysts, much higher than that of unsupported Ni, and the oscillating behaviour of Ni/Z_m_ (*red*) and Ni/Z_hy_ (*red*) catalysts suggest that the active sites are possibly Ni sites at the boundary between metal particles and the support. The electron density of these Ni sites is sufficiently affected by the interaction with zirconia and/or with nearby carbide-like species to modify the catalytic behaviour of these Ni sites with respect to unsupported Ni metal particles.

Furthermore, Ni active sites should only be a very small fraction of the boundary species, as TPR results indicated a markedly lower amount of interacting Ni species in the 5.1Ni/Z_m_ catalyst than in the 4.8Ni/Z_hy_ catalyst (11% vs 100%), both showing similar catalytic performance.

In the literature, the CH_4_-CPO mechanism was described as a direct oxidation route, requiring several Ni sites able to activate methane and oxygen in adjacent positions (Ni–C, Ni–O and Ni–H) [14,17,36,60]. According to this pathway, we suggest that the active sites for the CH_4_-CPO on Ni/ZrO_2_ catalysts should be poly-nuclear metal sites at the metal particle boundary, in a specific configuration. A similar proposal on the presence of cooperating nearby sites has been made to explain the catalytic activity of Ni supported on grafted ZrO_2_-Al_2_O_3_ catalysts [36]. The amount of poly-nuclear metal active sites was lower in the Ni/Z (*red*) than in the Ni/Z (*ox-red*) catalysts because, in the Ni/Z (*red*), some Ni^δ+^ carbide-like species formed, inducing CPO oscillation.

## 4. Conclusions

Ni metal particles supported on monoclinic ZrO_2_, prepared via the impregnation of two different starting materials, were active for the partial oxidation of CH_4_. As both monoclinic ZrO_2_ and unsupported Ni metal were inactive for the CH_4_-CPO under the same experimental conditions, the metal–support interaction played a key role in developing active Ni sites. Although the starting material strongly influenced the size of the supported NiO particles due to the different number of available anchoring sites, the NiO-ZrO_2_ interaction was strong enough to prevent the sintering of metal particles formed during the *ox-red* or *red* activation treatment. In the same way, the Ni-ZrO_2_ interaction was strong enough to guarantee the stability of metal particles during the catalytic runs.

Different activation treatments of catalysts yielded different catalytic properties: the *ox-red* treatment yielded nonoscillating activity and a selectivity of Ni sites, whereas the *red* treatment induced an oscillating behaviour. It can be suggested that the *red* activation treatment reduced NiO and carbonates adsorbed on the zirconia surface, leading to the formation of Ni^δ+^ carbide-like species. These Ni^δ+^ sites, having a greater tendency to change the oxidation state, were responsible for oscillations in the catalytic activity. Conversely, the *ox-red* treatment, removing adsorbed carbonates during the oxidation step, yielded Ni active sites with metal characteristics, producing a nonoscillating catalytic activity.

We conclude that Ni dispersion was not the main factor affecting the activity and that the active sites were a small fraction of all the exposed Ni atoms, likely those at the boundary of the metal particles in a specific configuration and nuclearity, strongly depending on the activation procedure. The interaction of these specific Ni atoms with the zirconia surface and/or with nearby carbide-like species was strong enough to modify the catalytic behaviour of the Ni species with respect to the unsupported Ni metal.

## Figures and Tables

**Table 1:** Structural and textural features of precursors and catalysts (activated or used): specific surface area (S.A.), total pore volume (V_tot_) and crystallite size (d_XRD_) of ZrO_2_, NiO and Ni.

Precursors	Catalysts	S.A. ^1^ (m^2^·g^−1^)	V_tot_ ^2^ (cc·g^−1^)	Crystallite Size, d_XRD_ (nm)			
				ZrO_2_	NiO	Ni act ^3^ Used	
Z_hy_	-	360	0.33	-	-	-	-
-	Z_m_	5.6	0.041	49	-	-	-
NiO (unsupported)	-	-	-	-	70	-	-
-	Ni (unsupported)	-	-	-	-	-	70
1.8NiO/Z_m_	-	2.7	0.024	-	22	-	-
-	1.8Ni/Z_m_(ox-red)	-	-	-	-	18	28
	1.8Ni/Z_m_(red)	-	-	-	-	25	-
5.1NiO/Z_m_	-	3.3	0.031	50	36	-	-
-	5.1Ni/Z_m_(ox-red)	-	-	-	-	30	25
	5.1Ni/Z_m_(red)	-	-	-	-	35	37
1.7NiO/Z_hy_	-	5.4	0.040		32	-	-
-	1.7Ni/Z_hy_(ox-red)	-	-	-	-	-	20
	1.7Ni/Z_hy_(red)	-	-	-	-	-	25
4.8NiO/Z_hy_	-	4.4	0.044	52	26	-	-
-	4.8Ni/Z_hy_(ox-red)	-	-	-	-	23	-
	4.8Ni/Z_hy_(red)	-	-	-	-	24	30

^1^ ±0.5 m^2^·g^-1^; ^2^ ±0.005 cm^3^·g^-1^; ^3^ act, activated catalysts.

**Table 2:** FESEM images analysis for Z_m_, NiO and Ni.

Precursors	Catalysts	Particle Size, d_FESEM_ (nm)				Ni Particle Number	Ni Particle Mean Diameter		Ni Dispersion	Exposed Ni Atoms
		ZrO_2_	NiO	Ni Activated Used		Σn_i_ ^1^	d_LN_ ^2^ (nm)	d_VA_ ^3^ (nm)	D ^4^ (%)	N_Ni(exp)_^5^ (atoms/g)
-	Z_m_	30–100	-	-	-	-	-	-	-	-
NiO (unsupported)	-	-	20–400	-	-	-	-	-	-	-
-	Ni (unsupported)			40–1500	50–2000	-	-	-	-	-
1.8NiO/Z_m_	-	-	40–60	-	-	-	-	-	-	-
-	1.8Ni/Z_m_ (*ox-red*)	-	-	5–75	20–80	1043	36 ± 15	48	2.1	3.9·10^18^
	1.8Ni/Z_m_ (*red*)	-	-	20–75	20–80	-	-	-	-	-
5.1NiO/Z_m_	-	-	80–300	-	-	-	-	-	-	-
-	5.1Ni/Z_m_ (*ox-red*)	-	-	30–1000	50–700	502	2.6·10^2^	5.5·10^2^	0.18	9.4·10^17^
	5.1Ni/Z_m_ (*red*)	-	-	30–700	35–700	-	-	-	-	-
1.7NiO/Z_hy_	-	-	n.d. ^6^	-	-	-	-	-	-	-
-	1.7Ni/Z_hy_ (*ox-red*)	-	-	10–35	15–25	25	18 ± 7	22	4.6	7.1·10^18^
	1.7Ni/Z_hy_ (*red*)	-	-	5–30	15–20	-	-	-	-	-
4.8NiO/Z_hy_	-	-	n.d. ^6^	-	-	-	-	-	-	-
-	4.8Ni/Z_hy_ (*ox-red*)	-	-	10–45	15–40	188	24 ± 7	28	3.6	1.8·10^19^
	4.8Ni/Z_hy_ (*red*)	-	-	8–35	15–40	-	-	-	-	-

^1^ n_i_, number of metal particles with a specific diameter d_i_; ^2^ length-number mean diameter, d_LN_ = Σn_i_·d_i_/Σn_i_ and the corresponding standard deviation σ; ^3^ volume-area mean diameter, d_VA_ = Σ_i_n_i_d_i_^3^/Σn_i_d_i_^2^; ^4^ Ni dispersion, D = 100 ·6⋅(*v*_Ni_/*a*_Ni_)/d_VA_; ^5^ exposed Ni atoms, N_Ni(exp)_ = (D·w_Ni_·N_A_)/(10,000·M_Ni_); ^6^ n.d., not detected.

**Table 3:** H_2_-TPR results for NiO and Ni/ZrO_2_ catalyst precursors.

Catalyst	H_2_ Consumption (μmoli g^−1^)	e/Ni ^1^	% NiO Species		
			α	β	γ
NiO	1330	2.00	100	-	-
1.8Ni/Z_m_	31.04	2.02	-	60	40
5.1Ni/Z_m_	97.07	2.03	89	4	7
1.7Ni/Z_hy_	29.93	2.02	-	54	46
4.8Ni/Z_hy_	83.75	1.97	-	68	32

^1^ Number of electrons consumed per Ni atom (e/Ni), calculated as (2·H_2_ molecules consumed g^−1^)/(Ni atoms·g^−1^).

## Data Availability

Data are contained within the article and supplementary material.

## Supplementary Materials

The following are available online at https://www.mdpi.com/article/10.3390/ma14102495/s1, Figure S1: Catalytic activity with contact time. Figure S2: Nitrogen adsorption/desorption isotherms (a) and pore size distributions (b) for the Z_m_ support and NiO/ZrO_2_ catalyst precursors. Figure S3: Reproducibility of activity in four subsequent runs on two representative samples activated by the *ox-red* treatment (a) or by the *red* treatment (b). Figure S4: Percent H_2_ selectivity (a) and percent CO selectivity (b) for Ni/ZrO_2_ catalysts after *red* activation treatment as a function of temperature. Figure S5: Catalytic activity of 5.1Ni/Z_m_ (*red*) catalyst at 923 K with different total flow rates. CH_4_ conversion and H_2_, CO_2_ and H_2_O yields as a function of time on stream. Figure S6: Catalytic activity of 1.8Ni/Z_m_ (*red*) catalyst at 1023 K with different O_2_ contents in the feed. CH_4_ conversion and H_2_, CO_2_ and H_2_O yields as a function of time on stream.
