# Supplementary material for: Oscillatory Behaviour of Ni Supported on ZrO2 in the Catalytic Partial Oxidation of Methane as Determined by Activation Procedure
Source: Materials (Basel). 2021 May 12;14(10):2495. doi: 10.3390/ma14102495 (PMC8150648; doi:10.3390/ma14102495)
Supplement: Supplementary file 1 [file materials-14-02495-s001.zip › materials-1186679-supplementary.pdf]

materials

Supplementary Material

Oscillatory Behaviour of Ni Supported on ZrO_2 in the Catalytic Partial Oxidation of Methane as Determined by Activation Procedure

Daniela Pietrogiacomi [1,*], Maria Cristina Campa [2], Ida Pettiti [1], Simonetta Tuti [3], Giulia Luccisano [1], Leandro Ardemani [1], Igor Luisetto [4] and Delia Gazzoli [1]

[1] Chemistry Department, Sapienza University of Rome, P.le Aldo Moro 5, 00185 Rome, Italy; ida.pettiti@uniroma1.it (I.P.); giulia.luccisano@uniroma1.it (G.L.); leandrodoc@yahoo.com (L.A.); delia.gazzoli@uniroma1.it (D.G.)

[2] Institute for the Study of Nanostructured Materials (ISMN), National Research Council (CNR), Sapienza University of Rome, P.le Aldo Moro 5, 00185 Rome, Italy; mariacristina.campa@cnr.it

[3] Science Department, Roma Tre University, Via della Vasca Navale 79, 00146 Rome, Italy; simonetta.tuti@uniroma3.it

[4] Italian National Agency for New Technologies, Energy and Sustainable Economic Development (ENEA), Casaccia Research Centre, Via Anguillarese 301, 00123 Rome, Italy; igor.luisetto@enea.it

* Correspondence: daniela.pietrogiacomi@uniroma1.it; Tel.: +39-06-49913304

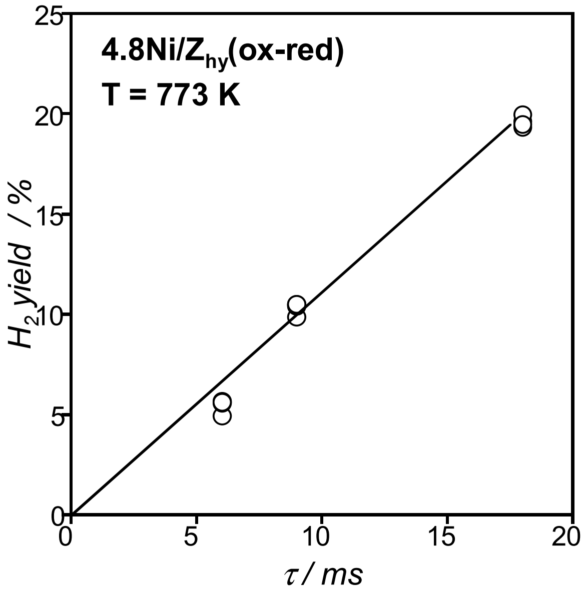

Figure S1. Catalytic activity with contact time. H_2 yield at 773 K with different amounts of 4.8Ni/Z_{hy}(*ox-red*) catalyst (30, 50 and 100 mg). Reactant mixture: [CH_4] = 2%, [O_2] = 1%, N_2 as balance; total flow rate = 150 cm^3 (STP)·min^{-1}. Contact time, τ = W/(F·d), where W is the sample-weight, F is the flow rate and d is the sample apparent bulk density (d = 2 g·cm^{-3}).

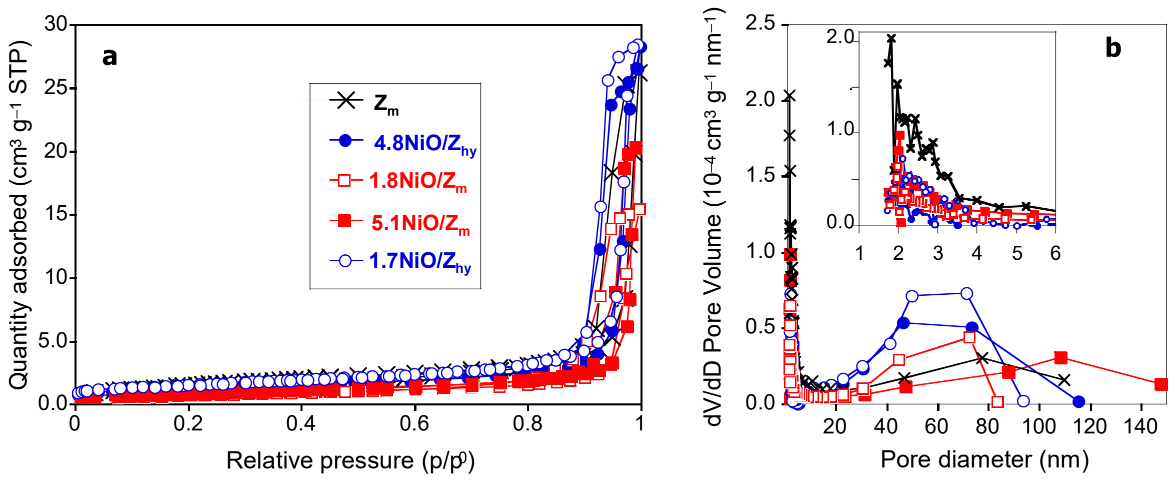

Figure S2. Nitrogen adsorption/desorption isotherms (**a**) and pore size distributions (**b**) for the Z_m support and NiO/ZrO_2 catalyst precursors.

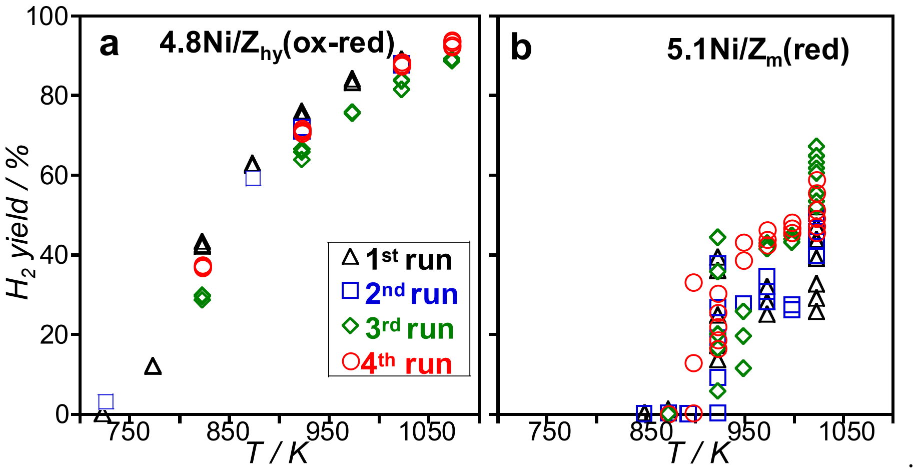

Figure S3. Reproducibility of activity in four subsequent runs on two representative samples activated by the *ox-red* treatment (**a**) or by the *red* treatment (**b**). H_2 yield as a function of temperature. Reactant mixture: [CH_4] = 2%, [O_2] = 1%, N_2 as balance; total flow rate = 150 cm³ (STP)·min⁻¹.

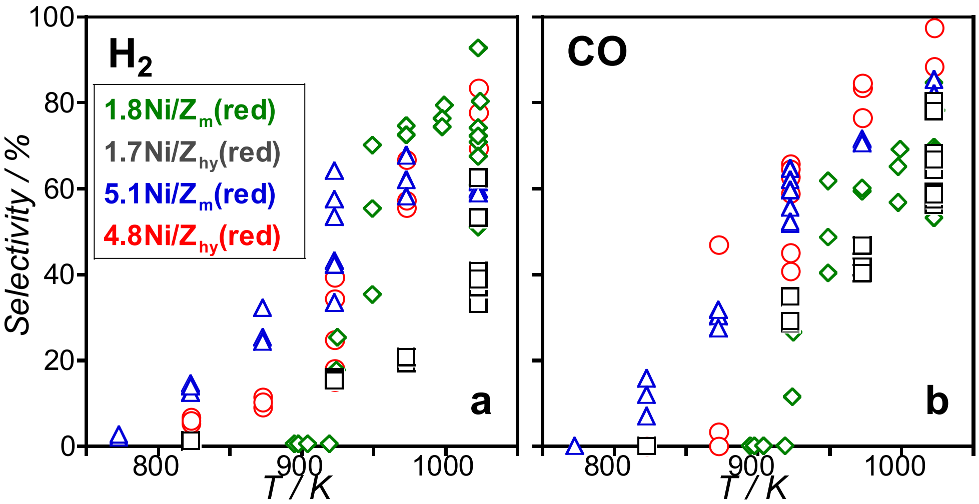

Figure S4. Percent H_2 selectivity (**a**) and percent CO selectivity (**b**) for Ni/ZrO_2 catalysts after *red* activation treatment as a function of temperature. Reactant mixture: [CH_4] = 2%, [O_2] = 1%, N_2 as balance; total flow rate = 150 cm³ (STP)·min⁻¹.

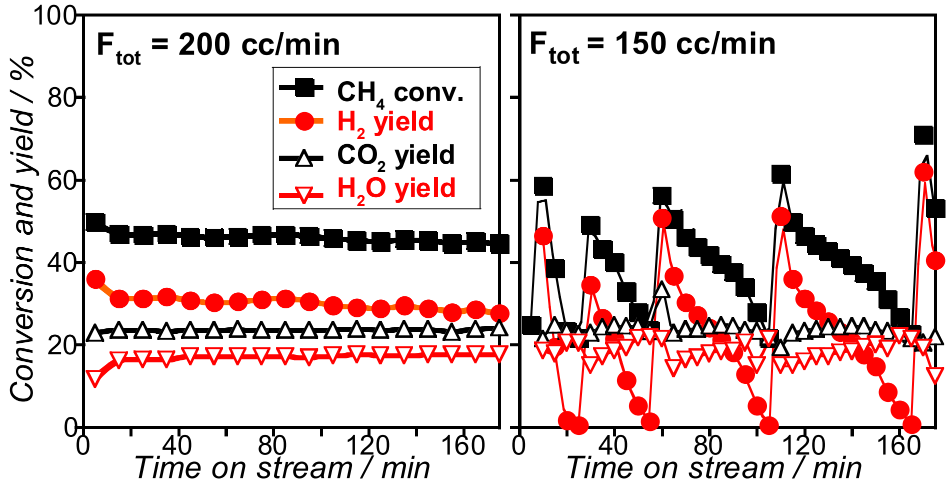

Figure S5. Catalytic activity of 5.1Ni/Z_m(*red*) catalyst at 923 K with different total flow rates. CH_4 conversion and H_2, CO_2 and H_2O yields as a function of time on stream. Reactant mixture: [CH_4] = 2%, [O_2] = 1%, N_2 as balance; total flow rate = 200 (**left**) or 150 (**right**) cm³ (STP)·min⁻¹.

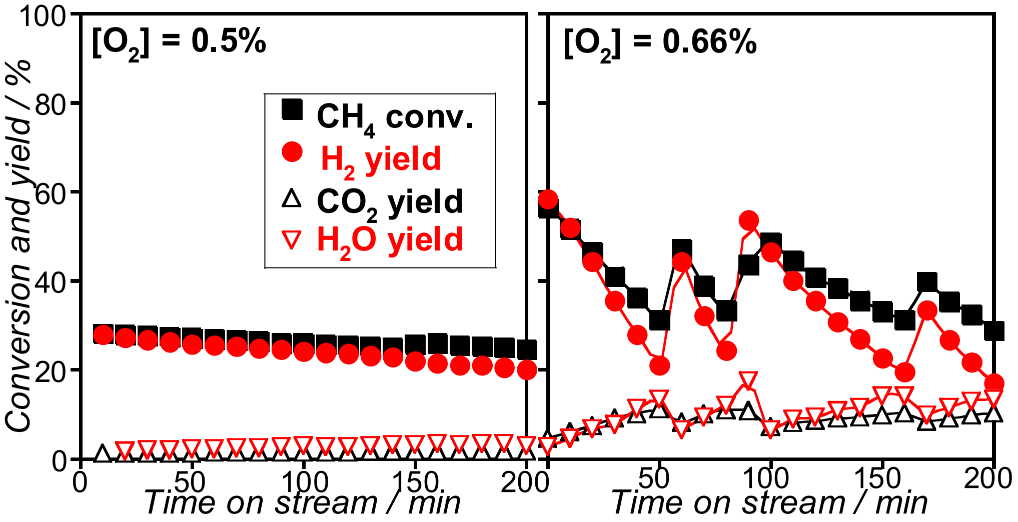

Figure S6. Catalytic activity of 1.8Ni/Zm(*red*) catalyst at 1023 K with different O_2 contents in the feed. CH_4 conversion and H_2, CO_2 and H_2O yields as a function of time on stream. Reactant mixture: [CH_4] = 2%, [O_2] = 0.5 (**left**) or 0.66% (**right**), N_2 as balance; total flow rate = 150 cm^3 (STP)·min^{-1}.
